# Xylo‐oligosaccharides as texture modifier compounds in aqueous media and in combination with food thickeners

**DOI:** 10.1002/fsn3.1177

**Published:** 2019-09-10

**Authors:** Péter Penksza, Réka Juhász, Beatrix Szabó‐Nótin, László Sipos

**Affiliations:** ^1^ Department of Food Preservation Szent István University Budapest Hungary; ^2^ Department of Dietetics and Nutrition Science, Faculty of Health Science Semmelweis University Budapest Hungary; ^3^ Department of Postharvest Sciences and Sensory Evaluation, Faculty of Food Science Szent István University Budapest Hungary

**Keywords:** fructo‐oligosaccharides, gelatin, locust bean gum, oscillatory, rotational rheological methods, xanthan gum, xylo‐oligosaccharides

## Abstract

Present study introduces the previously not‐described rheological properties of a nondigestible oligosaccharide: xylo‐oligosaccharide which is a novel food ingredient in Europe. Significant differences were observed among viscosity of solutions of different formulas (liquid or powder) of XOS. Thickening potential of XOS in aqueous media compared to that of sucrose (Suc) or fructo‐oligosaccharides strongly depends on utilization level: At low concentration, XOS proved to be weaker while at high concentration to be stronger than fructo‐oligosaccharides. Differences in viscosity of XOS, FOS, and sucrose were much higher at below 60°C than at higher temperatures. Storage and loss modulii of xanthan gum gels were not influenced while those of locust bean gum were affected negatively by XOS addition. Addition of XOS at low concentrations did not decrease gelatin gel strength but increased gelatin gel stability against mechanical stress. XOS proved to have different rheological behavior from previously used oligosaccharides.

## Graphical abstract

Present study introduces the previously not‐described rheological properties of a nondigestible oligosaccharide: xylo‐oligosaccharide. We investigated the thickening potential of XOS in aqueous media compared to that of sucrose and fructo‐oligosaccharides. Results indicated that texture‐modifying potential of XOS is different from previously used oligosaccharides, and therefore, it should be taken into account during food product development.

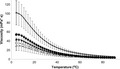

## 1 INTRODUCTION

Nondigestive oligosaccharides (NDOs) have resistance to human digestive enzymes in stomach and small intestine. The NDOs are claimed to behave as dietary fibers and prebiotics. Enrichment of diet with NDOs gives opportunity for improving gut microecology including bacterial populations, biochemical profiles, and physiological effects (Mussatto & Mancilha, 2007). Several NDOs are used in food industry (Sako, Matsumoto, & Tanaka, 1999), among them the most known are the fructo‐oligosaccharides (FOS) which are often used as sugar replacer on account of its sweet taste and as thickener in low‐fat products (Franck, 2002). A new type of NDOs is XOS which is well known and widely used as functional food ingredient or food supplement in Japan and in China. In Japan, XOS are approved as food ingredients by Foods for Specified Health Uses (FOSHU), specifically for foods to modify gastrointestinal conditions. In China, XOS commercialized since 2000 and used as food supplement and as a functional compound in dairy products (Makelainen, Juntunen, & Hasselwander, 2009). In Europe, XOS is a novel food ingredient, and our research team is working on the authorization according to suggestions of Regulation (EC) No 258/97 of the European Parliament and of the Council of 27 January 1997 concerning novel foods and novel food ingredients. Present study introduces the first steps of this work by testing the previously not‐described technological properties of XOS.

Xylo‐oligosaccharides are oligomers of two to ten β‐1, 4‐linked xylose monomers. XOS are hydrolysis products of xylan and are available as white powder or as liquid syrup. XOS are found in fruits, vegetables, bamboo, honey, milk, as well as in xylan‐rich lignocellulosic material obtained from agricultural, forestal, and industrial waste (Carvalho et al., 2015; Vázquez, Alonso, Domínguez, & Parajó, 2000). These oligosaccharides are stable at temperatures up to 100°C and over a wide pH range of 2.5–8.0 therefore in the gastric pH too (Courtin, Swennen, Verjans, & Delcour, 2009). XOS are nondigestible, noncariogenic prebiotics which stimulate bacterial growth and fermentation, improve intestinal mineral absorption, and also possess antioxidant effect (Moure, Gullón, Domínguez, & Parajó, 2006). Efficacious dose of XOS as prebiotics has been suggested to be as low as 1 g per day (Makelainen et al., 2009).

XOS products are available in syrup or powder form and are predominantly composed of the disaccharide xylobiose, the trisaccharide xylotriose, and the tetrasaccharide xylotetraose with small amounts of higher oligosaccharides also present. High‐purity XOS products contain at least 70%–95% XOS which are intended to use in food industry. XOS 70L syrup form is a transparent clear yellow‐colored, high‐viscosity liquid with no particles. XOS 70P and XOS 95P powder forms are white powders with good solubility in cold water. Little is known about the rheological properties of XOS. Park, Lee, Lee, Rhew, and Yang (2001) reported that the viscosity of 70Bx XOS proved to be 930cP at 20°C and 90cP at 60°C, which was higher than that of sugar but lower than that of other oligosaccharides. Mumtaz, Rehman, Huma, Jamil, and Nawaz (2008) observed that in yoghurts, XOS could be used up to 3.5% concentration level and that it shows strengthening effect on viscosity in combination with gelatin.

Aim of research was to investigate the rheological behavior of XOS to evaluate its potential texture‐modifying effect in order to establish food product development. Furthermore, to evaluate the differences between available XOS products (powder and liquid forms, purity of 70 or 95%), rheological behavior of different types of XOS in aqueous media was investigated by rotational and oscillatory methods:

1. in function of concentration and in comparison with fructo‐oligosaccharides (FOS) and sucrose;
2. in function of temperature between 4 and 90°C in order to evaluate effect of food processing technologies;
3. in combination with food thickeners widely used in food industry such as gelatin, xanthan gum, and locust bean gum.

## 2 MATERIALS AND METHODS

### 2.1 Materials

Three types of XOS (Longlive Shandong Co. Ltd): 95P (powder, contains 95% XOS), 70L (syrup, contains 70% XOS), 70P (powder, contains 70% XOS) and as control fructo‐oligosaccharide (FOS; syrup, contains 85% FOS, Orafti®, Beneo), and commercially available sucrose (Suc) was used in the experiment. In order to evaluate interaction with texture modifiers, oscillation rheological tests were performed using commercially available thickening agents: gelatin (GEL; commercially available, form pork, Bloom degree: 180, Hungary), xanthan gum (XG; Sigma‐Aldrich Hungary Ltd), and locust bean gum flour (LBG; Sigma‐Aldrich Hungary Ltd).

### 2.2 Sample preparation

Sample preparation is introduced according to the three main objectives of the study.

#### 2.2.1 Experiment 1

Rheological behavior of different types of XOS in aqueous media in function of concentration and in comparison with FOS and sucrose was investigated by rotational technique and referred in present study as Experiment 1. Thirteen aqueous solutions with 0.5, 1, 2, 3, 4, 5, 10, 20, 30, 40, 50, 60, and 70% dry material content were prepared of five samples (three types of XOS, sucrose, and FOS), and their flow curve was measured using five parallels.

#### 2.2.2 Experiment 2

Effect of temperature on the rheological behavior of oligosaccharides was tested by rotational technique at constant shear rate using 50% dry material content solution of five samples (XOS 70L, 70P, 95P, sucrose, and FOS).

#### 2.2.3 Experiment 3

Interaction between food thickeners and xylo‐oligosaccharides was evaluated in aqueous gels. Type and concentration of food thickeners were selected based on previous experiments with fruit jellies. Control samples were prepared by dissolving 1.0 g locust bean gum + 1.0 g xanthan gum or 5.0 g of gelatin in distilled water to get a final weight of 100.0 g. All types of XOS (70L, 70P, 95P) were added to each texture modifier in 1 and 3% concentration. 1.0 or 3.0 g of XOS, respectively, was mixed with texture modifier and then dissolved in distilled water to get a final weight of 100.0 g. Mixtures were heated up to 80°C and after 30 min cooled down to room temperature. Oscillatory measurements were performed after 24 hr rest at room temperature in order to let gel forming to occur.

### 2.3 Rheological measurements

The measurements were taken using Physica MCR 51 (Anton‐Paar, Anton‐Paar Hungary Ltd) rheometer. Results were recorded and analyzed using Rheoplus software 3.2. (Anton‐Paar, Anton‐Paar Hungary Ltd). Rotational measurements (Experiments 1 and 2) were performed using a concentric cylinder measuring system consisting of DG27 double‐gap cylindrical measuring bob 27 mm in diameter and C‐PTD cylinder. Oscillation tests (Experiment 3) were performed with a plate and plate (PP50/S: plate measuring bob 50 mm in diameter and P‐PTD200 plate) measuring system using 2 mm gap size.

#### 2.3.1 Experiment 1

Type and concentration effect were tested by recording the flow curve (shear stress in function of the shear rate) of the samples. Measurements were taken at 20°C, and the shear rate increased logarithmically between 1 and 1,200 1/sec during 160 s, recording 32 measuring points for each sample. Herschel–Bulkley model (Eq.1.) was fitted to the measurement results, and consistency (C, Pa.s), flow index (n), yield stress (τ_0_, Pa), and correlation coefficient (R^2^) between measured and calculated values were determined.

(1) $$\tau =\tau_{0}+C\cdot {\dot{\gamma }}^{n}$$

#### 2.3.2 Experiment 2

The viscosity in function of temperature was recorded during the test of temperature effect. The shear rate was constant at 400 1/s, and temperature increased in 4–90°C interval by a constant heating rate of 5°C/min, recording 50 measuring points. Five replicates were used.

#### 2.3.3 Experiment 3

Interaction between food thickeners and xylo‐oligosaccharides was evaluated using oscillatory technique. The amplitude sweep method was performed at 4°C, at constant angular frequency (10 rad/s), increasing strain logarithmically from 0.01% to 200% during 150 s and recording a measuring point at each 5 s (30 measured points per measurement). Five parallels were tested of each sample. Storage (G’, Pa) and loss modulus (G”, Pa) were recorded in function of shear stress (τ, Pa). Crossover (Cross, Pa) the intersection of storage and loss modulus curves, and initial G’ and G” values (G’_0_/G”_0_, dimensionless unite) were calculated during the evaluation of oscillation measurement data of the samples. The shear stress value at which the G’ decreases by 5% compared to the initial G' value is the end of the linear viscoelastic range, called LVE (Pa). The complex viscosity (Pa.s) was determined by the complex modulus divided by the angular frequency (Mezger, 2006).

### 2.4 Statistical evaluation

All of the measurements were taken in five replicates. Kruskal–Wallis nonparametric test was applied to calculate the exact *p*‐value (*α* = 0.05), and Dunn's pairwise procedure (post hoc test) was used with Bonferroni correction. The analyses were implemented by the XL‐Stat Sensory solution version 2013.1.01 software (Addinsoft, 28 West 27th Street, Suite 503, New York, NY 10001, USA).

## 3 RESULTS AND DISCUSSION

### 3.1 Experiment 1. Flow properties of xylo‐oligosaccharides in function of concentration and in comparison with fructo‐oligosaccharides and sucrose

Flow curve of aqueous solutions with different concentration levels of oligosaccharides was measured. Herschel–Bulkley model fitted well to the measured flow curves of aqueous solutions of XOS, FOS, and Suc as indicated by correlation coefficient values (0.995<*R* ^2 ^< 0.999). Power low index (n) values did not show significant difference from 1 and yield stress proved to be 0, indicating that all of the measured samples belong to the Newtonian fluids.

The consistency of the solution of the oligosaccharides in function of the concentration showed similar changes (Table 1).

**Table 1:** The consistency (C) values of different types of oligosaccharides with indication of their homogenous and heterogeneous groups tested by Kruskal–Wallis statistical test

Samples	Concentration (%)												
	0.5	1	2	3	4	5	10	20	30	40	50	60	70
Sucrose	0.25 ± 0.15^a^	0.19 ± 0.11^a^	0.22 ± 0.1^a^	0.43 ± 0.13^ab^	0.33 ± 0.25^a^	0.27 ± 0.15^a^	0.29 ± 0.1^ab^	1.6 ± 0.19^ab^	1.93 ± 0.2^a^	1.2 ± 0.03^a^	14.36 ± 9.53^abc^	89.29 ± 16.06^abc^	736.26 ± 9^ab^
Fructo‐oligosaccharides	2.9 ± 0.82^b^	4.48 ± 3.05^b^	3.76 ± 1.6^b^	3.5 ± 3.02^b^	2.95 ± 2.06^b^	2.22 ± 1.10^b^	2.39 ± 0.86^c^	3.67 ± 0.14^b^	5.51 ± 0.15^a^	4.21 ± 0.38^ab^	3.69 ± 1.38^a^	46.15 ± 6.81^a^	288.33 ± 32.11^a^
70L	0.43 ± 0.18^ab^	0.4 ± 0.27^ab^	0.52 ± 0.31^ab^	0.41 ± 5.80^b^	0.49 ± 3.65^ab^	0.74 ± 0.40^ab^	0.91 ± 0.19b^c^	1.21 ± 0.1^ab^	1.45 ± 0.11^a^	1.6 ± 0.23^a^	9.89 ± 4.22^ab^	54.75 ± 4.88^ab^	678.62 ± 19.1^a^
70P	0.17 ± 0.09^a^	0.2 ± 0.07^a^	0.15 ± 0.07^a^	0.26 ± 0.14^a^	0.25 ± 0.18^a^	0.23 ± 0.16^a^	0.28 ± 0.07^a^	1.01 ± 0.01^a^	4.2 ± 0.41^a^	20.0 ± 3.87^b^	44.6 ± 12.03^c^	165.42 ± 45.5^c^	1,444.94 ± 16.67^c^
95P	0.43 ± 0.23^ab^	0.38 ± 0.17^ab^	0.38 ± 0.1^ab^	0.57 ± 0.3^ab^	0.56 ± 0.3^ab^	0.64 ± 0.4^ab^	0.35 ± 0.07^abc^	1.89 ± 0.13^ab^	7.86 ± 1.28^a^	7.9 ± 4.79^ab^	38.25 ± 6.19^bc^	113.84 ± 23.25^bc^	1,278.12 ± 208.4^bc^

Note

xylo‐oligosaccharides in liquid form with 70% XOS content, 70P: xylo‐oligosaccharides in powder form with 70% XOS content, 95P: xylo‐oligosaccharides in powder form with 95% XOS content.

^a,b,c^indicate 95% confidence level.

The values of consistency slightly increased until 50% concentration and then sharply increased until 70%. The values of consistency (C) of different types of oligosaccharides were compared to each other at all of the thirteen concentration levels tested by Kruskal–Wallis statistical test. C values with indication of their homogenous groups are shown in Table 1.

In the first interval at low concentration (between 0.5% and 4% dry material content), consistency of FOS was significantly higher than that of three types of XOS and of sucrose and was no significant difference among the latter. Results indicate that in liquid food products and at low concentration, xylo‐oligosaccharides possess similar thickening effect as sucrose and lower than FOS. It suggests that XOS could be effectively used to replace bulking effect of sucrose in low‐calorie beverages in combination with intensive sweeteners.

In the second interval between 4% and 40%, dry material content consistency of powder type XOS products (95P and 70P) increased more intensively than that of the other three oligosaccharides (FOS, 70L, and sucrose). Significant differences were observed among consistency of 70P and other oligosaccharides. 30% dry material content was special because there was no significant difference between the materials tested. At this concentration level, flow curves showed a slight change in slope at 600 1/s shear rate and higher standard deviation on consistency than at other concentration levels. Results suggest that there is a change in hydration mechanism of oligosaccharides at low and high concentration resulted in a marked change of rheological properties. In the fourth interval at high concentration (50%–70%), consistency of XOS, 70P, and 95P solutions was significantly higher than that of the other oligosaccharides. It may be caused by differences in water‐binding mechanism of powder form products compared to liquid form or by differences in molecular weight distribution. The 70L and the FOS were already in liquid state, not bind as much of water; hence, these samples had minor viscosity increasing effect. Results indicated that thickening potential of tested oligosaccharides strongly depends on the level of use: At low concentration, FOS proved to be the most effective but at high concentration XOS showed better thickening potential. Furthermore, it seems that there is a difference in water‐binding mechanism of different types of oligosaccharides and during food product development it should be considered and recipes changed accordingly.

### 3.2 Experiment 2. Effect of temperature on flow properties of xylo‐oligosaccharides in comparison with fructo‐oligosaccharides and sucrose

**Figure 1:**
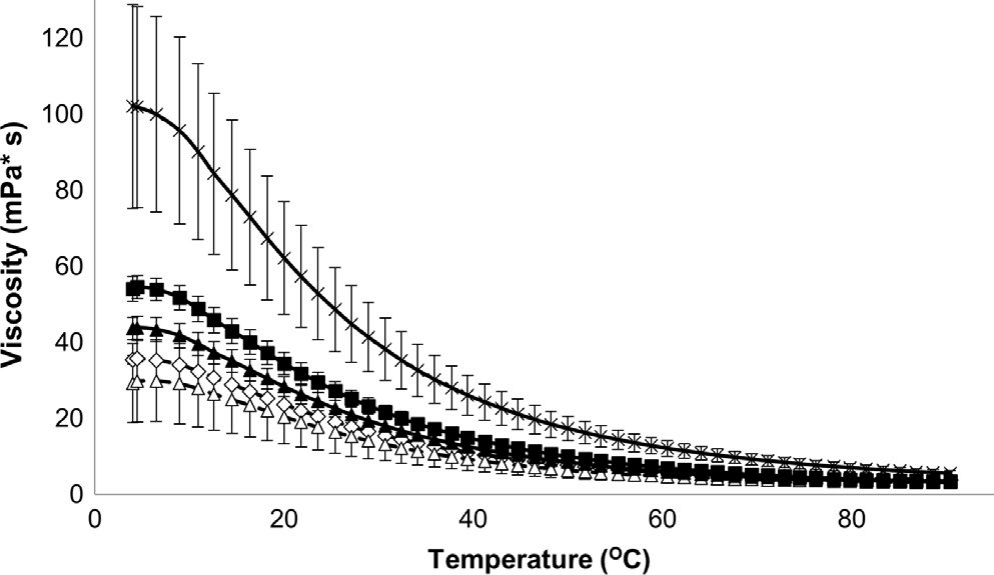
Viscosity of xylo‐oligosaccharides, fructo‐oligosaccharides, and sucrose in function of temperature. 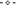
Sucrose; 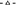
FOS; 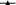
70L; 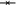
70P; 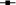
95P

Viscosity of three types of XOS, FOS, and Suc in function of temperature is shown in Figure 1 and was measured in order to evaluate the effect of possible food processing technologies on the rheological behavior of oligosaccharides. The viscosity values of different types of oligosaccharides were compared to each other at 50 temperature levels tested by Kruskal–Wallis statistical method. Viscosity values with indication of their homogenous groups and further detailed statistical results are shown in Table S1.

The viscosity of all oligosaccharides except for XOS 70P was stable between 4 and 10°C and then decreased until the end of the measurement. Decrease in viscosity was more intensively between 10 and 50°C than between 50 and 90°C. Viscosity of XOS70P proved to be the highest and that of FOS to be the lowest in the whole temperature range investigated. Differences between viscosity values of five types of oligosaccharides tested decreased as temperature increased. Viscosity of XOS 70P proved to be significantly higher than that of FOS between 4 and 90°C and that of sucrose between 4 and 60°C.

According to Park et al. (2001), the viscosity of XOS is more stable against temperature than that of sucrose. Our results indicated that though viscosity values of XOS samples were higher than of sucrose and of the FOS, the viscosity of sucrose and FOS was more resistant to temperature increasing. Either at refrigerator or at room temperature (e.g., in yoghurts, beverages), the XOS increases the viscosity of a foodstuff more than FOS or sucrose, but at higher temperatures (e.g., in ready‐to‐eat meals or custards), there is no significant difference between the texture modifier effects of oligosaccharides tested.

### 3.3 Experiment 3. Interaction between xylo‐oligosaccharides and other texture modifiers

#### 3.3.1 Gelatin

Interaction between XOS and widely used texture modifiers was investigated in aqueous gels using XOS at different concentration levels. Mumtaz et al. (2008) observed that in yoghurts XOS could be used up to 3.5% concentration level and that it shows strengthening effect on viscosity in combination with gelatin.

**Figure 2:**
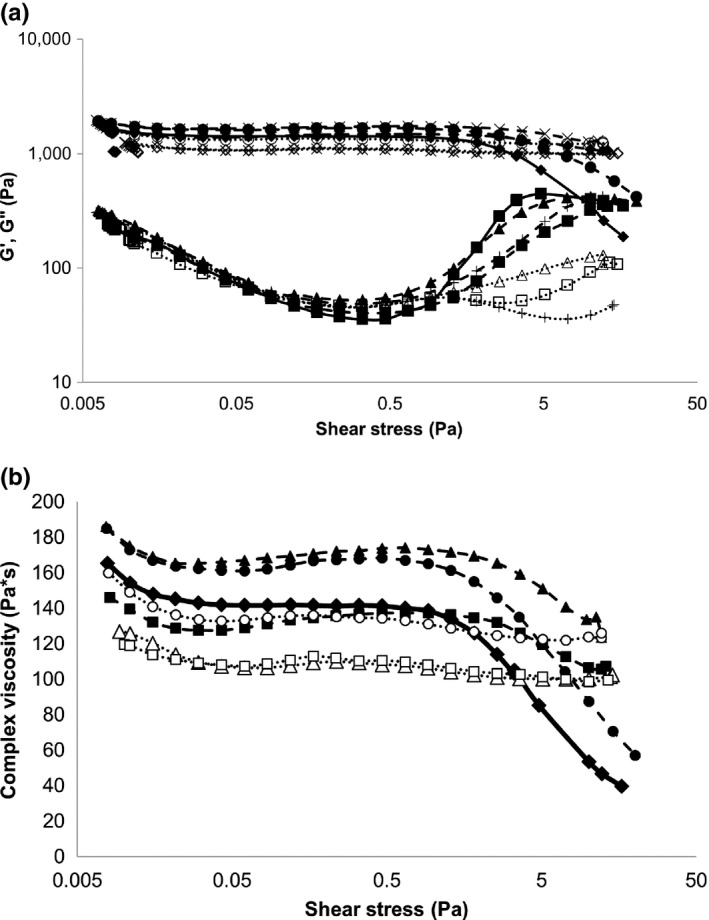
(a) Amplitude sweep rheograms of gelatin gels prepared with or without xylo‐oligosaccharide addition. 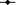
G′ 0%; 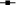
G″ 0%; 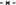
G′ 1%‐70L; 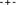
G″ 1%‐70L; 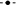
G′ 1%‐70P; 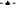
G″ 1%‐70P; 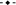
G′ 1%‐95P; 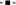
G″ 1%‐95P; 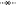
G′ 3%‐70L; 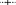
G″ 3%‐70L; 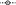
G′ 3%‐70P; 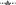
G″ 3%‐70P; 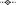
G′ 3%‐95P; 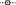
G″ 3%‐95p. (b) Complex viscosity of gelatin gels prepared with or without xylo‐oligosaccharide addition. 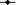
0%; 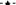
1%‐70L; 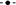
1%‐70P; 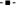
1%‐95P; 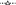
3%‐70L; 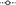
3%‐70P; 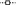
3%‐95P

Amplitude sweep rheogram of gelatin gels are shown in Figure 2a and complex viscosity of gelatin gels in function of shear stress in Figure 2b. The G’ and G” values of gelatin gels with different types of xylo‐oligosaccharides were compared to each other at 31 shear stress values by Kruskal–Wallis statistical test method. The G’ and G” values of gelatin gels with different types of xylo‐oligosaccharides were compared to each other at 31 shear stress values by Kruskal–Wallis statistical test method. G’ and G” values with indication of their homogenous groups and further detailed statistical results are shown in Table S2.

G’ values varied in the range 1000–2000 Pa, while G” values between 35 and 444 Pa. G’ to G” ratio was in the range 1–10, indicating that a gel was formed (Mezger, 2006). The shape of rheogram was similar in case of all samples prepared with XOS addition. Crossover was observed solely on rheogram of control gelatin gel. G’ was constant up to shear stress 10 Pa in all samples and then slightly decreased in case of control, 70P 1%, and 95P 1%. G” decreased continuously in 10^–2^–10^0^ Pa shear stress interval. Beyond 10^0^ Pa shear stress, G” of gelatin gels with 0 and 1% XOS decreased intensely while G” of gelatin gels with 3% XOS increased slightly.

Ratio of initial storage and loss moduli (G’_0_ and G”_0_) (Table 2) of control and XOS‐containing gelatin gels showed no significant difference, indicating that XOS addition did not affect the resting gel strength. However, lack of crossover of XOS‐containing samples suggests that XOS addition increased mechanical stability of gelatin gel.

**Table 2:** Amplitude sweep parameters of control and XOS‐containing food additives gels with indication of their homogenous and heterogeneous groups tested by Kruskal–Wallis statistical test

Xylo‐ oligosaccharides type		Locust bean gum			Locust bean gum + xanthan gum			Xanthan gum			Gelatin
	level	LVE	Cross	G'_0_/G"_0_	LVE	Cross	G'_0_/G"_0_	LVE	Cross	G'_0_/G"_0_	G'_0_/G"_0_
–	0%	53 ± 1.61^c^	316 ± 15.17^c^	1.32 ± 0.015^b^	97 ± 3.46^c^	840 ± 48.17^c^	6.22 ± 0.75^c^	12 ± 4.58^a^	55 ± 2.53^abc^	2.74 ± 0.31^a^	22.17 ± 5.68^a^
70L	1%	30 ± 1.02^abc^	187 ± 6.99^ab^	1.15 ± 0.032^a^	54 ± 3.91^abc^	446 ± 18.22^a^	4.19 ± 0.64^abc^	16 ± 4.71^abc^	54 ± 2.58^abc^	2.60 ± 0.25^a^	23.44 ± 4.60^c^
	3%	25 ± 0.21^a^	148 ± 3.49^a^	1.10 ± 0.04^a^	50 ± 2.13^a^	456 ± 24.34^ab^	4.78 ± 0.32^abc^	32 ± 0.96^bc^	57 ± 0.36^bc^	2.63 ± 0.25^a^	15.39 ± 0.68^ab^
70P	1%	31 ± 2.64^abc^	195 ± 5.07^abc^	1.18 ± 0.037^ab^	54 ± 6.63^abc^	495 ± 70.71^ab^	3.86 ± 0.31^ab^	21 ± 6.64^abc^	54 ± 0.11^ab^	2.68 ± 0.15^a^	21.55 ± 0.61^bc^
	3%	29 ± 0.93^ab^	186 ± 1.94^ab^	1.16 ± 0.034^ab^	53 ± 1.10^ab^	521 ± 45.41^abc^	3.68 ± 0.16^a^	14 ± 2.81^bc^	52 ± 0.67^a^	2.46 ± 0.37^a^	19.36 ± 4.19^abc^
95P	1%	40 ± 2.42^bc^	252 ± 7.80^bc^	1.22 ± 0.093^ab^	65 ± 1.07^bc^	605 ± 46.70^abc^	4.04 ± 0.22^abc^	15 ± 2.34^ab^	56 ± 0.72^abc^	2.43 ± 0.10^a^	20.80 ± 1.40^bc^
	3%	34 ± 2.42^bc^	229 ± 8.37^bc^	1.28 ± 0.034^b^	73 ± 5.31^bc^	656 ± 83.63^bc^	5.34 ± 0.41^bc^	24 ± 7.43^c^	58 ± 0.42^c^	2.56 ± 0.08^a^	19.36 ± 3.53^abc^

Note

LVE: end of the linear viscoelastic range (Pa); cross: crossover (Pa) the intersection of storage and loss modulus curves; G'_0_/G"_0_: initial G’(storage moduli, Pa) and G” (loss moduli, Pa) ratio; 70L: xylo‐oligosaccharides in liquid form with 70% XOS content, 70P: xylo‐oligosaccharides in powder form with 70% XOS content, 95P: xylo‐oligosaccharides in powder form with 95% XOS content.

^a,b,c^indicate 95% confidence level.

Complex viscosity of gelatin gels in function of shear stress (Figure 2b) of gelatin gels with 1% XOS was higher in 0.01–1 Pa shear stress interval than that of samples with 3% XOS. Above 1 Pa shear stress complex viscosity of control sample decreased intensely while that of samples with 1% XOS decreased slightly. Complex viscosity of samples with 3% XOS did not change above 1 Pa shear stress. The 3% 70L had the largest value of storage modulus, while the other two 3% sample showed smaller values than the other three 1% XOS contained samples. Thus, the bigger amount of water contained gels formed the more plastic gelatin gels. Decreasing slope of complex viscosity curves in range 1–10 Pa shear stress in function of increasing amount of XOS (Table 2 **)** suggests that stability against mechanical stress increased because of addition of XOS.

Our results indicate that XOS addition at low concentration results a stronger initial gel (using more water resulting stronger gel), while the higher XOS concentration results a gel more stable against increasing shear stress. However, other oligosaccharides (sugars, i.e., sucrose and polyols) have been reported to increase the gel strength of 7% (w/w) gelatin gels (Kasapis & Al‐Marhoobi, 2003) which could be explained by that sugars and polyols are excluded from the domain of the protein thus the protein–sugar interface reducted which stabilize the protein gel system (Gekko & Timasheff, 1981; Kasapis & Al‐Marhoobi, 2005).

#### 3.3.2 Carbohydrate‐based food additives

**Figure 3:**
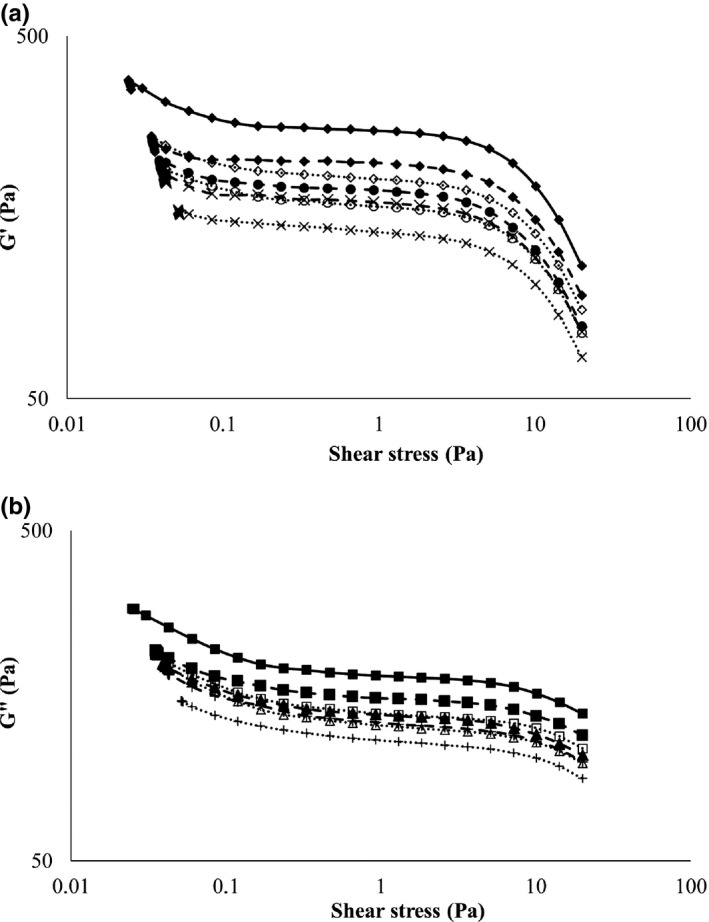
(a) G’ rheograms of locust bean gum gels prepared with or without xylo‐oligosaccharide addition. 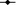
G′ 0%; 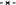
G′ 1%‐70L; 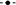
G′ 1%‐70P; 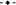
G′ 1%‐95P; 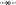
G′ 3%‐70L; 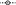
G′ 3%‐70P; 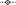
G′ 3%‐95P. (b) G” rheograms of locust bean gum gels prepared with or without xylo‐oligosaccharide addition. 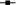
G″ 0%; 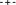
G″ 1%‐70L; 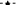
G″ 1%‐70P; 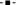
G″ 1%‐95P; 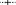
G″ 3%‐70L; 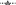
G″ 3%‐70P; 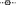
G″ 3%‐95P

Amplitude sweep rheograms of locust bean gum gels with or without XOS addition and those of LBG are shown in Figure 3a,b. The G’(Figure 3a) and G” (Figure 3b) values of LBG or xanthan gels with different types of xylo‐oligosaccharides were compared to each other at 31 shear stress values by Kruskal–Wallis statistical test method. The G’ and G” values of LBG or xanthan gels with different types of xylo‐oligosaccharides were compared to each other at 31 shear stress values by Kruskal–Wallis statistical test method. G’ and G” values of LBG, XG, and LBG + XG gels with indication of their homogenous and heterogeneous groups and further detailed statistical results are shown in Table S3, Table S4, and Table S5, respectively.

### 3.4 Locust bean gum

Amplitude sweep rheograms of locust bean gum gels with or without XOS addition showed similar shape (Figure 3a,b) with decreasing G’ and G” between shear stress of 0.1–10 Pa and a crossover between 10 and 100 Pa. G’ and G” values were in the same range between 60 and 400 Pa. XOS‐containing samples showed lower G’ and G” values than control. Ratio of initial storage and loss moduli (G’_0_ and G”_0_) of samples with XOS95P proved to be the same as that of control (1.32), while in case of 70L and 70P samples, it was significantly lower (Table 2). Decrease in G’_0_ to G”_0_ ratio indicates decrease in hardness at low deformation force. LVE and crossover parameters proved to be significantly lower in case of XOS‐containing LBG gels than in control (Table 2), indicating decreased stability of LBG gel structure because of XOS addition. The XOS cannot interact with the LBG; however, the water is distracted away from the solution and hence the LBG decreases the ability to enforce the texture properties. XOS has the same effect on the gels as well as the sucrose by binding water thus delay gelatinization (BeMiller, 2007).

### 3.5 Xanthan

Amplitude sweep rheograms of xanthan gum gels (Figure 3a,b) showed similar shape with slightly decreasing G’ values between 0.1 and 2.5 Pa share stress than an intense decrease in G’ values followed by a crossover. Rheograms showed similar G’ and G” values (in the range of 10–200 Pa). Because of the higher amount presence of maltodextrin in 3% 70P, which also binds large quantities of water, therefore a weaker gel was formed in the presence of the xanthan. G’ to G” ratio and crossover of the XOS‐containing xanthan gels did not show significant difference compared to control. (Table 2) LVE parameter also showed similar values in all of the xanthan gels tested except for sample with XOS 70L 3%. LVE value of the latter proved to be higher than that of control. Addition of XOS did not influence hardness or stability of the xanthan gels. Hence, the xanthan is charged, and due to the side chains are polared, it is an easily soluble material, and different oligosaccharide concentrations do not affect the ability of gelatinization. Furthermore, in such small amounts of XOS, it did not show the effect of increasing the gel viscosity.

### 3.6 Locust bean gum + Xanthan gum

Similar tendencies have been observed in case of gels with LBG and XG combination as in case of gels prepared with LBG alone. Rheograms showed similar shape with G’ values between 110 and 1,450 Pa and G” values between 100 and 400 Pa and with a crossover point at 15 Pa shear stress. Control showed higher G’ and G” values, higher initial G’ to G” ratio, higher LVE, and crossover values than XOS‐containing LBG + XG gels (Table 2). As excepted, 70L 3% and 95P 3% gels showed similar initial hardness to control. These results indicate that addition of XOS to LBG + XG gels affected negatively hardness and stability.

XOS addition affected negatively gel strength and stability of LBG and LBG + XG gels. In case of XG, the large amount of maltodextrin containing 70P sample had negative effects on the felling properties. Therefore be said that XOS water‐binding capacity reduces the LBG gel formatting ability, while the xanthan gum is not sensitive to the 1 and 3% concentrations except if the sample contains larger quantities of maltodextrin. That is, we have to be considered that the form and the composition of XOS should impact on the texture modifiers. This is related to the low molecular weight compounds such as sucrose reported to decrease intristic viscosity of LBG gels (Elfak, Pass, Phillips, & Morley, 1977) because of either a reduction in solvent quality or decrease in polymer/polymer association (Richardson, Willmer, & Foster, 1998).

## 4 CONCLUSIONS

Based on the rheological measurement of aqueous solutions, different formulas of xylo‐oligosaccharides XOS 70P proved to have significantly higher consistency than 70L or 95P. At low concentrations, fructo‐oligosaccharides showed the highest viscosity, while XOS 70L and 95P were the same as sucrose. However, increasing concentration above 40(w/w) % FOS showed lower viscosity than 70P due to changes of the hydration mechanism.

At low temperature range (4–60°C), bigger differences were observed among viscosity of oligosaccharides than at higher temperatures (60–90°C). Indicating that in refrigerated food products such as yoghurts, texture‐modifying effect of XOS has primary importance.

Addition of XOS at low concentrations (1–3 w/w%) did not decrease gelatin gel strength as indicated by ratio of initial storage modulus (G’) and storage modulus (G”) values but increased gelatin gel stability against mechanical stress as indicated by decreasing slope of complex viscosity with increasing amount of XOS. Addition of XOS at low concentrations did not influence gel properties (storage modulus (G’) and storage modulus (G”), crossover or linear viscoelastic range) of xanthan gels. However, presence of XOS weakened gel strength of gels prepared with locust bean gum alone or in combination with xanthan.

Results indicated that texture‐modifying potential of XOS is different from previously used oligosaccharides (FOS or Suc), and therefore, it should be taking into account during food product development.

## CONFLICT OF INTEREST

The authors declare that they do not have any conflict of interest.

## ETHICAL APPROVAL

This study does not involve any human or animal testing.

## References

[fsn31177-bib-0002] BeMiller, J. N. (2007). Carbohydrate chemistry for food scientist (2nd edn). St. Paul, MN: AACC International.

[fsn31177-bib-0003] Carvalho, A. F. A. , Oliva‐Neto, P. , de Almeida, P. Z. , da Silva, J. B. , Escaramboni, B. , & Pastore, G. M. (2015). Screening of xylanolytic *Aspergillus fumigatus* for prebiotic xylo‐oligosaccharide production using bagasse. Food Technology and Biotechnology, 53, 428–435. 10.17113/ftb.53.04.15.4160 27904377 PMC5079171

[fsn31177-bib-0004] Courtin, C. M. , Swennen, K. , Verjans, P. , & Delcour, J. A. (2009). Heat and pH stability of prebiotic arabinoxylooligosaccharides, xylooligosaccharides and fructooligosaccharides. Food Chemistry, 112, 831–837. 10.1016/j.foodchem.2008.06.039

[fsn31177-bib-0006] Elfak, A. M. , Pass, G. , Phillips, G. O. , & Morley, R. G. (1977). The viscosity of dilute solutions of guar gum and locust bean gum with and without added sugars. Journal of the Science of Food and Agriculture, 28, 895–899. 10.1002/jsfa.2740281005

[fsn31177-bib-0007] Franck, A. (2002). Technological functionality of inulin and oligofructose. British Journal of Nutrition, 87, 287–291. 10.1079/bjnbjn/2002550 12088531

[fsn31177-bib-0008] Gekko, K. , & Timasheff, S. N. (1981). Mechanism of protein stabilization by glycerol: Preferential hydration in glycerol‐water mixtures. Biochemistry, 20, 4667–4676. 10.1021/bi00519a023 7295639

[fsn31177-bib-0009] Kasapis, S. , & Al‐Marhoobi, I. M. (2005). Bridging the divide between the high‐ and low‐solid analyses in the gelatin/κ‐carrageenan mixture. Biomacromolecules, 6, 14–23. 10.1021/bm0400473 15638497

[fsn31177-bib-0010] Kasapis, S. , Al‐Marhoobi, I. M. , Deszczynski, M. , Mitchell, J. R. , & Abeysekera, R. (2003). Gelatin vs polysaccharide in mixture with sugar. Biomacromolecules, 4, 1142–1149. 10.1021/bm0201237 12959577

[fsn31177-bib-0011] Makelainen, H. , Juntunen, M. , & Hasselwander, O. (2009). Prebiotic potential of xylo‐oligosaccharides In Charalampopoulos D. & Rastall A. (Eds.), Prebiotics and probiotics science and technology (pp. 245–258). New York, NY: Springer Science.

[fsn31177-bib-0012] Mezger, T. G. (2006). The rheology handbook, (2nd edn). Hannover: Vincentz Network.

[fsn31177-bib-0013] Moure, A. , Gullón, P. , Domínguez, H. , & Parajó, J. C. (2006). Advances in the manufacture. purification and applications of xylo‐oligosaccharides as food additives and nutraceuticals. Process Biochemistry, 41, 1913–1923. 10.1016/j.procbio.2006.05.011

[fsn31177-bib-0014] Mumtaz, S. , Rehman, U. S. , Huma, N. , Jamil, A. , & Nawaz, H. (2008). Xylooligosaccharide enriched yoghurt: Physicochemical and sensory evaluation. Pakistan Journal of Nutrition, 7, 566–569. 10.3923/pjn.2008.566.569

[fsn31177-bib-0015] Mussatto, S. I. , & Mancilha, I. M. (2007). Non‐digestible oligosaccharides: A review. Carbohydrate Polymers, 68, 587–597. 10.1016/j.carbpol.2006.12.011

[fsn31177-bib-0016] Park, Y. J. , Lee, J. W. , Lee, C. S. , Rhew, B. K. , & Yang, C. K. (2001). Physicochemical properties of xylooligosaccharide as food material. Korean Journal of Food Science and Technology, 33(1), 19–23.

[fsn31177-bib-0017] Richardson, P. H. , Willmer, J. , & Foster, T. J. (1998). Dilute solution properties of guar and locust bean gum in sucrose solutions. Food Hydrocolloid, 12, 339–348. 10.1016/S0268-005X(98)00025-3

[fsn31177-bib-0018] Sako, T. , Matsumoto, K. , & Tanaka, R. (1999). Recent progress on research and applications of non‐digestible galacto‐oligosaccharides. International Dairy Journal, 9, 69–80. 10.1016/S0958-6946(99)00046-1

[fsn31177-bib-0019] Vázquez, M. J. , Alonso, J. L. , Domínguez, H. , & Parajó, J. C. (2000). Xylo‐oligosaccharides: Manufacture and applications. Trends in Food Science & Technology, 11, 387–393. 10.1016/S0924-2244(01)00031-0

