# Supplementary material for: Xylo‐oligosaccharides as texture modifier compounds in aqueous media and in combination with food thickeners
Source: Food Sci Nutr. 2019 Sep 10;8(7):3023–30. doi: 10.1002/fsn3.1177 (PMC7382132; doi:10.1002/fsn3.1177)
Supplement: Supplementary file 1 [file FSN3-8-3023-s001.docx]

Table S1.**.** Viscosity values of xylo-oligosaccharides, fructo-oligosaccharides and sucrose in function of temperature

| **Temperature °C** | **Samples** | | | | |
| --- | --- | --- | --- | --- | --- |
|  | Sucrose | Fructo-  oligosaccharides | 70L | 70P | 95P |
| 4.0 | 35.46±1.12^ab^ | 29.26±10.45^a^ | 43.62±2.3^abc^ | 102.12±26.79^c^ | 54.1±3.34^bc^ |
| 4.5 | 35.68±0.72^ab^ | 29.82±10.72^a^ | 43.84±3.05^abc^ | 101.88±16.4^c^ | 54.52±3.04^bc^ |
| 6.48 | 35.32±0.32^a^ | 29.92±10.8^a^ | 43.36±3.19^ab^ | 100±25.72^b^ | 53.96±3.01^ab^ |
| 8.96 | 34.08±0.26^a^ | 29.12±10.58^a^ | 41.74±3.16^ab^ | 95.68±24.58^b^ | 51.74±3.2^ab^ |
| 10.9 | 32.38±0.28^a^ | 27.84±10.09^a^ | 39.56±3.03^ab^ | 90.2±23.06^b^ | 48.8±3.32^ab^ |
| 12.6 | 30.66±0.33^a^ | 26.4±9.51^a^ | 37.28±2.96^ab^ | 84.36±21.19^b^ | 45.88±3.33^ab^ |
| 14.5 | 28.9±0.44^a^ | 24.94±8.9^a^ | 35.04±2.9^ab^ | 78.76±19.72^b^ | 43.02±3.27^ab^ |
| 16.4 | 27.1±0.46^a^ | 23.44±8.33^a^ | 32.7±2.82^ab^ | 72.96±17.82^b^ | 40.08±3.23^ab^ |
| 18.2 | 25.28±0.45^a^ | 21.92±7.75^a^ | 30.48±2.71^ab^ | 67.42±16.29^b^ | 37.2±3.11^ab^ |
| 20.0 | 23.62±0.52^a^ | 20.38±7.15^a^ | 28.32±2.61^ab^ | 62.18±14.81^b^ | 34.44±2.99^ab^ |
| 21.8 | 22.02±0.52^a^ | 18.98±6.55^a^ | 26.28±2.49^ab^ | 57.32±13.42^b^ | 31.88±2.79^ab^ |
| 23.6 | 20.5±0.48^a^ | 17.66±6.03^a^ | 24.38±2.36^ab^ | 52.76±12.15^b^ | 29.5±2.6^ab^ |
| 25.4 | 19.08±0.52^a^ | 16.4±5.54^a^ | 22.6±2.23^ab^ | 48.62±11^b^ | 27.26±2.41^ab^ |
| 27.1 | 17.8±0.48^a^ | 15.2±5.1^a^ | 20.96±2.14^ab^ | 44.84±10.01^b^ | 25.2±2.24^ab^ |
| 28.9 | 16.58±0.48^a^ | 14.14±4.7^a^ | 19.42±2.01^ab^ | 41.38±9.06^b^ | 23.32±2.08^ab^ |
| 30.7 | 15.44±0.44^a^ | 13.14±4.33^a^ | 18±1.93^ab^ | 38.2±8.22^b^ | 21.6±1.93^ab^ |
| 32.4 | 14.42±0.41^a^ | 12.22±3.98^a^ | 16.74±1.83^ab^ | 35.28±7.5^b^ | 20±1.78^ab^ |
| 34.1 | 13.44±0.39^a^ | 11.37±3.70^a^ | 15.54±1.73^ab^ | 32.66±6.87b | 18.6±1.63^ab^ |
| 35.9 | 12.58±0.36^a^ | 10.6±3.43^a^ | 14.46±1.65^ab^ | 30.24±6.27^b^ | 17.26±1.46^ab^ |
| 37.7 | 11.78±0.33^a^ | 9.91±3.2^a^ | 13.48±1.58^ab^ | 28.06±5.69^b^ | 16.06±1.37^ab^ |
| 39.4 | 11.02±0.31^a^ | 9.23±2.95^a^ | 12.56±1.48^ab^ | 26.08±5.21^b^ | 14.94±1.22^ab^ |
| 41.2 | 10.38±0.28^a^ | 8.65±2.79^a^ | 11.72±1.43^ab^ | 24.28±4.78^b^ | 13.92±1.14^ab^ |
| 43.0 | 9.76±0.26^a^ | 8.07±2.59^a^ | 10.95±1.34^ab^ | 22.62±4.39^b^ | 13±1.06^ab^ |
| 44.7 | 9.18±0.27^a^ | 7.59±2.45^a^ | 10.28±1.31^ab^ | 21.1±4.04^b^ | 12.08±1.04^ab^ |
| 46.5 | 8.66±0.27^a^ | 7.12±2.3^a^ | 9.63±1.26^ab^ | 19.72±3.72^b^ | 11.32±0.92^ab^ |
| 48.2 | 8.166±0.25^a^ | 6.69±2.17^a^ | 9.03±1.21^ab^ | 18.46±3.45^b^ | 10.61±0.87^ab^ |
| 50.0 | 7.718±0.25^a^ | 6.298±2.04^a^ | 8.482±1.17^ab^ | 17.32±3.22^b^ | 9.986±0.79^ab^ |
| 51.8 | 7.3±0.2^a^ | 5.94±1.93^a^ | 7.98±1.14^ab^ | 16.24±2.97^b^ | 9.370.7^ab^ |
| 53.5 | 6.94±0.27^a^ | 5.63±1.81^a^ | 7.51±1.11^ab^ | 15.28±2.76^b^ | 8.82±0.65^ab^ |
| 55.3 | 6.6±0.26^a^ | 5.34±1.7^a^ | 7.07±1.08^ab^ | 14.4±2.56^b^ | 8.3±0.58^ab^ |
| 57.0 | 6.27±0.26^a^ | 5.1±1.42^a^ | 6.68±1.06^ab^ | 13.56±2.39^b^ | 7.82±0.51^ab^ |
| 58.8 | 5.99±0.29^a^ | 4.87±1.43^a^ | 6.31±1.03^ab^ | 12.8±2.18^b^ | 7.39±0.509^ab^ |
| 60.5 | 5.72±0.27^a^ | 4.69±1.31^a^ | 5.97±1.01^ab^ | 12.1±2.07^b^ | 6.98±0.47^ab^ |
| 62.3 | 5.46±0.26^a^ | 4.5±1.22^a^ | 5.65±1^ab^ | 11.46±1.93^b^ | 6.6±0.43^ab^ |
| 64.1 | 5.21±0.27^a^ | 4.34±1.16^a^ | 5.35±0.98^ab^ | 10.85±1.85^b^ | 6.24±0.4^ab^ |
| 65.8 | 5.01±0.56^a^ | 4.16±1.06^a^ | 5.09±0.96^ab^ | 10.3±1.71^b^ | 5.92±0.38^ab^ |
| 67.6 | 4.84±0.28^ab^ | 4.02±1^a^ | 4.85±0.94^ab^ | 9.77±1.6^b^ | 5.6±0.35^ab^ |
| 69.4 | 4.73±0.3^ab^ | 3.93±0.98^a^ | 4.61±0.92^ab^ | 9.29±1.47^b^ | 5.32±0.33^ab^ |
| 71.1 | 4.64±0.3^ab^ | 3.81±0.94^a^ | 4.39±0.91^ab^ | 8.84±1.37^b^ | 5.05±0.31^ab^ |
| 72.9 | 4.54±0.25^ab^ | 3.74±0.95^a^ | 4.2±0.88^ab^ | 8.43±1.32^b^ | 4.82±0.3^ab^ |
| 74.5 | 4.48±0.26^ab^ | 3.64±0.94^a^ | 4.05±0.83^ab^ | 8.05±1.25^b^ | 4.59±0.27^ab^ |
| 76.2 | 4.38±0.3^ab^ | 3.58±0.90^a^ | 3.93±0.78^ab^ | 7.7±1.19^b^ | 4.38±0.27^ab^ |
| 78.1 | 4.3±0.32^ab^ | 3.5±0.9^a^ | 3.83±0.76^ab^ | 7.35±1.13^b^ | 4.18±0.25^ab^ |
| 79.8 | 4.22±0.32^ab^ | 3.41±0.89^a^ | 3.75±0.76^ab^ | 7.03±1.06^b^ | 4.02±0.23^ab^ |
| 81.5 | 4.14±0.33^ab^ | 3.35±0.85^a^ | 3.68±0.76^ab^ | 6.76±1.01^b^ | 3.87±0.19^ab^ |
| 83.3 | 4.05±0.32^ab^ | 3.28±0.82^a^ | 3.6±0.76^ab^ | 6.47±0.95^b^ | 3.76±0.18^ab^ |
| 85.1 | 3.99±0.31^ab^ | 3.23±0.82^a^ | 3.52±0.77^ab^ | 6.22±0.9^b^ | 3.66±0.17^a^ |
| 86.8 | 3.91±0.34^ab^ | 3.16±0.82^a^ | 3.45±0.76^ab^ | 5.98±0.85^b^ | 3.58±0.174^a^ |
| 88.6 | 3.84±0.33^ab^ | 3.11±0.82^a^ | 3.37±0.76^ab^ | 5.76±0.79^b^ | 3.53±0.18^a^ |
| 90.4 | 3.78±0.31^ab^ | 3.06±0.82^a^ | 3.31±0.75^ab^ | 5.56±0.72^b^ | 3.478±0.17^a^ |
