# Supplementary material for: Xylo‐oligosaccharides as texture modifier compounds in aqueous media and in combination with food thickeners
Source: Food Sci Nutr. 2019 Sep 10;8(7):3023–30. doi: 10.1002/fsn3.1177 (PMC7382132; doi:10.1002/fsn3.1177)
Supplement: Supplementary file 2 [file FSN3-8-3023-s002.docx]

Table S2. Storage and loss moduli values of gelatin gels prepared with or without xylo-oligosaccharides addition

|  | **0** | | | **95P 1%** | | **70P 1%** | | **70L 1%** | | **95P 3%** | | **70P 3%** | | **70L 3%** | |
| --- | --- | --- | --- | --- | --- | --- | --- | --- | --- | --- | --- | --- | --- | --- | --- |
| **Strain (%)** | G' | G" | G' | | G" | G' | G" | G' | G" | G' | G" | G' | G" | G' | G" |
| 0.0868 | 1633.33  ±  158.22^ab^ | 224.33  ±  57.06^ab^ | 1575  ±  263.88^ab^ | | 216.25  ±  48.73^ab^ | 1830  ±  190^b^ | 289  ±  33.18^b^ | 1840  ±  95.39^b^ | 263.33  ±  63.96^b^ | 1180  ±  95.39^a^ | 176  ±  32.45^a^ | 1576.67  ±  170.1^ab^ | 270.33  ±  68.97^b^ | 1260  ±  51.96^a^ | 210.67  ±  33.65^ab^ |
| 0.109 | 1530  ±  209.52^bc^ | 188.67  ±  30.73^ab^ | 1507.5  ±  251.18^abc^ | | 183  ±  36.94^a^ | 1713.33  ±  172.43^c^ | 235  ±  24.58^b^ | 1736.67  ±  90.74^c^ | 222.67  ±  52.31^ab^ | 1176.67  ±  85.05^a^ | 162.67  ±  23.69^a^ | 1470  ±  183.58^abc^ | 223.33  ±  51.73^b^ | 1223.33  ±  35.12^ab^ | 189.67  ±  31.5^ab^ |
| 0.153 | 1473.33  ±  206^ab^ | 164  ±  41.51^ab^ | 1435  ±  247.45^ab^ | | 157.5  ±  22.69^ab^ | 1660  ±  167.03^b^ | 183.67  ±  17.01^b^ | 1676.67  ±  58.59^b^ | 180.67  ±  46.2^b^ | 1133.33  ±  92.92^a^ | 134  ±  19.08^a^ | 1400  ±  191.57^ab^ | 177.67  ±  37.42^b^ | 1153.33  ±  37.86^a^ | 160  ±  21.38^ab^ |
| 0.215 | 1446.67  ±  219.39^ab^ | 129  ±  30.61^ab^ | 1400  ±  245.09^ab^ | | 125.5  ±  14.66^ab^ | 1630  ±  157.16^b^ | 142.67  ±  14.22^b^ | 1650  ±  43.59^b^ | 141.67  ±  37.54^b^ | 1104.33  ±  100.98^a^ | 106.53  ±  7.03^a^ | 1356.67  ±  193.99^ab^ | 137.67  ±  25.58^ab^ | 1100  ±  51.96^a^ | 129.67  ±  11.85^ab^ |
| 0.303 | 1426.67  ±  219.39^ab^ | 100.03  ±  19.2^abc^ | 1390  ±  246.58^ab^ | | 99  ±  9.67^ab^ | 1620  ±  157.16^b^ | 113.33  ±  11.85^bc^ | 1650  ±  30^b^ | 111.33  ±  28.29^c^ | 1089  ±  112.97^a^ | 89.17  ±  10.2^a^ | 1333.33  ±  188.24^ab^ | 107.13  ±  16.34^abc^ | 1076.67  ±  74^a^ | 105.6  ±  8.85^abc^ |
| 0.427 | 1420  ±  216.33^ab^ | 79.67  ±  12.06^a^ | 1392.5  ±  247.44^ab^ | | 78.75  ±  7.11^a^ | 1610  ±  153.94^b^ | 91.13  ±  8.55^b^ | 1653.33  ±  20.82^b^ | 88.87  ±  22.66^ab^ | 1075.67  ±  122.13^a^ | 75.53  ±  8.81^a^ | 1326.67  ±  193.99^ab^ | 86.03  ±  11.23^ab^ | 1061.67  ±  57.95^a^ | 84.3  ±  3.9^ab^ |
| 0.601 | 1416.67  ±  208.41^ab^ | 67.77  ±  9.3^ab^ | 1405  ±  248.93^ab^ | | 64.7  ±  5.76^a^ | 1610  ±  158.75^b^ | 74.7  ±  7.13^b^ | 1666.67  ±  15.28^b^ | 73.3  ±  16.73^ab^ | 1071.33  ±  129.02^a^ | 64.07  ±  7.12^a^ | 1333.33  ±  197.32^ab^ | 69.6  ±  6.08^ab^ | 1059.67  ±  61.4^a^ | 68.87  ±  3.58^ab^ |
| 0.846 | 1416.67  ±  196.04^ab^ | 54.1  ±  7.23^ab^ | 1427.5  ±  255^ab^ | | 55.23  ±  5^a^ | 1620  ±  190.79^b^ | 55.34  ±  9.27^ab^ | 1683.33  ±  31.15^b^ | 62.1  ±  15.27^ab^ | 1084  ±  140.81^a^ | 55.93  ±  4.42^ab^ | 1346.67  ±  195.02^ab^ | 59.77  ±  3.16^b^ | 1068.33  ±  63.71^a^ | 57.93  ±  2.69^ab^ |
| 1.19 | 1420  ±  185.2^ab^ | 46.67  ±  6.12^a^ | 1450  ±  267.46^ab^ | | 48.3  ±  4.7^a^ | 1643.33  ±  231.8^b^ | 58.23  ±  6.84^b^ | 1693.33  ±  41.63^b^ | 56.03  ±  11.84^b^ | 1104.33  ±  150.15^a^ | 51.2  ±  3.93^ab^ | 1356.67  ±  185.83^ab^ | 52.5  ±  3.12^ab^ | 1080  ±  55.68^a^ | 51.83  ±  2.95^ab^ |
| 1.67 | 1413.33  ±  171.56^ab^ | 40.77  ±  4.66^a^ | 1465  ±  286.65^ab^ | | 44.25  ±  5.36^a^ | 1666.67  ±  232.45^b^ | 54.77  ±  6.5^b^ | 1706.67  ±  49.33^b^ | 51.37  ±  9.23^ab^ | 1124.67  ±  159.52^a^ | 48.57  ±  4.42^ab^ | 1360  ±  164.62^ab^ | 47.93  ±  2.5^ab^ | 1096.67  ±  55.08^a^ | 47.87  ±  3.61^ab^ |
| 2.35 | 1413.33  ±  168.03^ab^ | 37.27  ±  3.35^a^ | 1475  ±  283.14^ab^ | | 41.63  ±  5.53^ab^ | 1673.33  ±  217.33^b^ | 52.5  ±  5.7^c^ | 1720  ±  43.59^b^ | 48.93  ±  7.3^bc^ | 1117.67  ±  160.95^a^ | 46.63  ±  3.26^bc^ | 1353.33  ±  161.66^ab^ | 45.4  ±  3.48^abc^ | 1093.33  ±  60.28^a^ | 45.93  ±  3.31^abc^ |
| 3.31 | 1416.67  ±  162.58^ab^ | 35.43  ±  2.8^a^ | 1480  ±  272.4^ab^ | | 40.38  ±  5.38^ab^ | 1673.33  ±  197.32^b^ | 52.37  ±  4.67^c^ | 1723.33  ±  28.87^b^ | 48.5  ±  6.92^bc^ | 1101.67  ±  160.96^a^ | 46  ±  3.58^abc^ | 1346.67  ±  158.85^ab^ | 45.2  ±  4.5^abc^ | 1083.33  ±  70.24^a^ | 45.53  ±  3.56^abc^ |
| 4.66 | 1413.33  ±  158.22^ab^ | 35.67  ±  3.56^a^ | 1485  ±  282.08^ab^ | | 40.48  ±  6.11^a^ | 1680  ±  200.75^b^ | 54.7  ±  6.15^b^ | 1733.33  ±  30.55^b^ | 50.27  ±  7.6^b^ | 1103.67  ±  167.48^a^ | 48  ±  4.56^ab^ | 1343.33  ±  153.08^ab^ | 47.3  ±  5.46^ab^ | 1080  ±  70^a^ | 47.5  ±  4.28^ab^ |
| 6.57 | 1400  ±  137.48^ab^ | 42.03  ±  8.87^a^ | 1487.5  ±  278.37^ab^ | | 42.18  ±  6.76^a^ | 1670  ±  190.79^b^ | 61.27  ±  11.91^b^ | 1740  ±  40^b^ | 53.43  ±  8.64^b^ | 1096.33  ±  172.98^a^ | 51.4  ±  6.07^ab^ | 1326.67  ±  141.54^ab^ | 50.9  ±  6.24^ab^ | 1069.33  ±  66.64^a^ | 51.37  ±  5.22^ab^ |
| 9.24 | 1386.67  ±  128.58^ab^ | 50  ±  22.57^a^ | 1482.5  ±  272.81^ab^ | | 47.08  ±  8.01^a^ | 1646.67  ±  176.16^b^ | 74.5  ±  25.81^b^ | 1730  ±  36.06^b^ | 61.77  ±  12.82^b^ | 1080.33  ±  186.128^a^ | 54.7  ±  7.67^ab^ | 1306.67  ±  133.17^ab^ | 56  ±  4.68^ab^ | 1050.67  ±  64.51^a^ | 54.57  ±  5.71^ab^ |
| 13 | 1333.33  ±  92.92^ab^ | 87.4  ±  38.43^b^ | 1470  ±  268.82^ab^ | | 56.55  ±  11.12^a^ | 1610  ±  156.21^b^ | 99  ±  50.75^b^ | 1716.67  ±  37.86^b^ | 74.63  ±  23.52^b^ | 1060.33  ±  196.19^a^ | 54.87  ±  6.71^abc^ | 1286.67  ±  133.17^ab^ | 61.43  ±  2.61^abc^ | 1027  ±  60.56^a^ | 54.63  ±  3.45^abc^ |
| 18.3 | 1250  ±  45.83^ab^ | 151  ±  75.02^b^ | 1447.5  ±  262.35^ab^ | | 76.33  ±  15.69^ab^ | 1543.33  ±  111.5^b^ | 150.87  ±  97.1^b^ | 1693.33  ±  40.42^b^ | 94.03  ±  39.04^ab^ | 1043  ±  201.86^a^ | 52.03  ±  3.19^a^ | 1263.33  ±  127.41^ab^ | 67.6  ±  13.3^ab^ | 1010.67  ±  64.51^a^ | 50.73  ±  0.95^a^ |
| 25.8 | 1096.67  ±  30.55^ab^ | 282  ±  137.12^c^ | 1402.5  ±  255.13^bc^ | | 111.7  ±  25.89^abc^ | 1436.67  ±  45.09^bc^ | 219.67  ±  145.23^c^ | 1650  ±  60.83^c^ | 125.43  ±  59.55^bc^ | 1031.67  ±  203.98^ab^ | 49.47  ±  1.27^ab^ | 1246.67  ±  133.17^abc^ | 75.83  ±  25.53^abc^ | 1003  ±  67.13^a^ | 45.3  ±  0.46^a^ |
|  |  |  |  | |  |  |  |  |  |  |  |  |  |  |  |
| 36.3 | 951.33  ±  71.99^a^ | 387  ±  134.38^c^ | 1335  ±  245.83^abc^ | | 157.25  ±  37.21^abc^ | 1303.33  ±  73.71^bc^ | 306  ±  191.28^c^ | 1576.67  ±  106.93^c^ | 176.37  ±  74.75^bc^ | 1024.67  ±  205.29^ab^ | 51.2  ±  7.51^ab^ | 1233.33  ±  144.68^abc^ | 86.17  ±  36.41^abc^ | 997.67  ±  69.33^ab^ | 40.27  ±  0.74^a^ |
| 50.9 | 973.33  ±  136.05^a^ | 394  ±  78.1^b^ | 1257.5  ±  247.98^ab^ | | 204.5  ±  38.72^ab^ | 1122  ±  161.78^ab^ | 370  ±  188.03^b^ | 1490  ±  151^b^ | 253.67  ±  73.06^ab^ | 1011.33  ±  204.51^a^ | 57.9  ±  14.95^a^ | 1216.67  ±  158.48^ab^ | 98.33  ±  45.65^ab^ | 997.33  ±  69.87^a^ | 36.87  ±  1.12^a^ |
| 71.9 | 717.33  ±  196.02^a^ | 444  ±  69^c^ | 1171.25  ±  256.66^abc^ | | 256  ±  41.46^abc^ | 938.33  ±  212.86^ab^ | 412  ±  142.97^c^ | 1363.33  ±  185.56^c^ | 343  ±  39.04^bc^ | 994.33  ±  199.74^abc^ | 70.43  ±  26.62^ab^ | 1216.67  ±  185.83^bc^ | 111.43  ±  43.81^abc^ | 1001.33  ±  73.38^abc^ | 35.9  ±  1.35^a^ |
| 102 | 343.33  ±  178.4^a^ | 399.67  ±  73.55^bc^ | 1091.75  ±  276.72^abc^ | | 318.5  ±  42.9^abc^ | 755.33  ±  208.06^ab^ | 419  ±  84.12^c^ | 1273.33  ±  277.55^c^ | 382  ±  51.29^bc^ | 980.67  ±  204.7^bc^ | 91.13  ±  44.8^ab^ | 1226.67  ±  234.59^c^ | 124.33  ±  39.95^abc^ | 1003  ±  70.76^abc^ | 38.73  ±  1.53^a^ |
| 142 | 258.33  ±  63.52^a^ | 386  ±  16.7^bc^ | 1064.25  ±  391.53^abc^ | | 357.5  ±  39^abc^ | 573  ±  163.33^ab^ | 400  ±  38.3^c^ | 1294  ±  369.69^bc^ | 367.67  ±  75.51^abc^ | 985  ±  231.1^abc^ | 110.6  ±  63.22^ab^ | 1224.67  ±  255.94^c^ | 129.23  ±  43.77^abc^ | 1018  ±  73.02^abc^ | 46.33  ±  5.49^a^ |
| 197 | 187.67  ±  21.55^a^ | 349.67  ±  14.22^abc^ | 1075.25  ±  530.07^bc^ | | 344.25  ±  36.99^abc^ | 417  ±  162.63^ab^ | 382.67  ±  46.76^bc^ | 1157  ±  315.37^c^ | 421.5  ±  67.18^c^ | 1004  ±  259.05^abc^ | 106.97  ±  55.3^ab^ | 1255  ±  360.62^bc^ | 107.2  ±  36.49^ab^ | 1024.67  ±  73.11^abc^ | 47.73  ±  5.56^a^ |
