# Supplementary material for: Xylo‐oligosaccharides as texture modifier compounds in aqueous media and in combination with food thickeners
Source: Food Sci Nutr. 2019 Sep 10;8(7):3023–30. doi: 10.1002/fsn3.1177 (PMC7382132; doi:10.1002/fsn3.1177)
Supplement: Supplementary file 3 [file FSN3-8-3023-s003.docx]

Table S3. Storage and loss moduli values of locust bean gum gels prepared with or without xylo-oligosaccharides addition

|  | 0% | | 95P 1% | | 70P 1% | | 70L 1% | | 95P 3% | | 70P 3% | | 70L 3% | |
| --- | --- | --- | --- | --- | --- | --- | --- | --- | --- | --- | --- | --- | --- | --- |
| Strain (%) | G' | G" | G' | G" | G' | G" | G' | G" | G' | G" | G' | G" | G' | G" |
| 0.601 | 311  ±  7.93^b^ | 23  5± | 231.33± | 190.67± | 210  ± | 177.7  ± | 193  ± | 168.33± | 233.67± | 183.33± | 202.67  ± | 175  ± | 161.33± | 146.33± |
|  |  | 8.71^b^ | 12.34^ab^ | 12.22^ab^ | 10.44^ab^ | 14.01^ab^ | 6^ab^ | 6.81^ab^ | 3.79^ab^ | 6.66^ab^ | 1.53^ab^ | 6.08^ab^ | 7.23^a^ | 11.7^a^ |
| 0.847 | 297.33± | 218.67± | 228  ± | 180.67± | 201  ± | 167.33± | 183.67± | 158  ± | 223.67± | 171.67± | 193.33  ± | 162± | 155.6± | 138± |
|  | 6.81^b^ | 7.02^b^ | 7.93^ab^ | 9.61^ab^ | 4.36^ab^ | 9.29^ab^ | 4.16^ab^ | 4.36^ab^ | 2.08^ab^ | 4.16^ab^ | 2.52^ab^ | 1.73^ab^ | 4.12^a^ | 7.81^a^ |
| 1.19 | 288  ± | 206  ± | 228.33± | 174.67± | 197.33± | 158.67± | 182  ± | 152  ± | 217  ± | 161.67± | 186  ±  5.2^ab^ | 151.67± | 153.67± | 132  ± |
|  | 7^b^ | 7^b^ | 3.51^ab^ | 6.03^ab^ | 1.53^ab^ | 4.04^ab^ | 4.58^ab^ | 4.36^ab^ | 1^ab^ | 3.21^ab^ |  | 1.54^ab^ | 3.79^a^ | 5.29^a^ |
| 1.68 | 282.33± | 196.67± | 227.33± | 169  ± | 195  ± | 153  ± | 182.33± | 147.33± | 212.33± | 154.67± | 180.33± | 143.33± | 152± | 127.67± |
|  | 6.42^b^ | 6.66^b^ | 0.58^ab^ | 3^ab^ | 6.56^ab^ | 4.36^ab^ | 4.16^ab^ | 3.21^ab^ | 3.21^ab^ | 3.51^ab^ | 5.03^ab^ | 2.31^ab^ | 4.35^a^ | 5.13^a^ |
| 2.36 | 280.67± | 191.67± | 226  ± | 164.33± | 192.33± | 148  ± | 179.33± | 141.67± | 209.67± | 149.67± | 176.67± | 138.33± | 150.33± | 124.33± |
|  | 4.93^b^ | 4.93^b^ | 1.73^ab^ | 2.52^ab^ | 7.02^ab^ | 4.36^ab^ | 2.31^ab^ | 2.08^ab^ | 4.04^ab^ | 2.52^ab^ | 5.31^ab^ | 1.53^ab^ | 3.21^a^ | 4.72^a^ |
| 3.32 | 279.67± | 189  ± | 225.33±  3.79^ab^ | 161  ± | 190.67± | 144  ± | 177  ± | 137  ± | 207.67± | 146.33± | 176  ± | 135.67± | 149  ± | 121.67± |
|  | 2.52^b^ | 3.46^b^ |  | 2.65^ab^ | 5.03^ab^ | 3.61^ab^ | 1^ab^ | 1^ab^ | 4.16^ab^ | 2.08^ab^ | 1.73^ab^ | 0.58^ab^ | 2.65^a^ | 3.51^a^ |
| 4.67 | 277.33± | 185.67± | 226  ± | 159  ± | 190  ± | 141.67± | 177  ± | 135  ± | 205.67± | 144  ± | 173.33  ± | 132.67± | 147.6  ± | 119.33± |
|  | 1.53^b^ | 3.21^b^ | 3.46^ab^ | 2^ab^ | 6^ab^ | 2.89^ab^ | 1^ab^ | 1^ab^ | 6.11^ab^ | 2.65^ab^ | 1.53^ab^ | 1.54^ab^ | 2.89^a^ | 3.06^a^ |
| 6.58 | 276.33± | 183.67± | 224  ± | 156.67± | 189  ± | 140  ± | 175.67± | 132.67± | 203.67± | 141.67± | 171.33  ± | 130.67± | 145.67± | 117.33± |
|  | 0.58^b^ | 2.31^b^ | 3.46^ab^ | 1.53^ab^ | 5.58^ab^ | 2.65^ab^ | 1.53^ab^ | 1.54^ab^ | 6.05^ab^ | 2.52^ab^ | 1.52^ab^ | 1.54^ab^ | 2.08^a^ | 2.08^a^ |
| 9.26 | 274.33± | 181.67± | 223.67± | 155.33± | 188  ± | 138.6  ± | 174.33± | 131.33± | 201.67± | 139.67± | 169.66  ± | 128.67± | 144  ± | 116  ± |
|  | 1.15^b^ | 1.53^b^ | 4.04^ab^ | 2.08^ab^ | 5^ab^ | 3.06^ab^ | 1.55^ab^ | 0.58^ab^ | 6.51^ab^ | 2.51^ab^ | 1.54^ab^ | 1.54^ab^ | 1.73^a^ | 2^a^ |
| 13 | 272.67± | 180.67± | 221.33± | 154.67± | 186  ± | 137.3  ± | 172.67± | 129.33± | 200  ± | 138.33± | 168  ± | 127.33± | 142.33± | 114.67± |
|  | 1.52^b^ | 1.53^b^ | 4.62^ab^ | 2.31^ab^ | 4.58^ab^ | 2.52^ab^ | 1.53^ab^ | 0.58^ab^ | 6.56^ab^ | 3.06^ab^ | 1^ab^ | 0.58^ab^ | 2.31^a^ | 1.52^a^ |
| 18.3 | 269.67± | 179  ± | 218.67± | 153.67±  2.31^ab^ | 183.67± | 136  ±  3^ab^ | 170.33± | 128.33± | 197.67± | 137  ± | 166  ± | 125.67± | 140.6  ± | 113.67± |
|  | 1.52^b^ | 1^b^ | 4.04^ab^ |  | 4.16^ab^ |  | 1.55^ab^ | 0.58^ab^ | 7.09^ab^ | 3^ab^ | 1^ab^ | 0.58^ab^ | 2.08^a^ | 1.51^a^ |
| 25.8 | 264.67± | 178  ± | 214.33± | 152  ± | 180  ± | 135  ± | 167  ± | 127  ± | 193.67± | 136  ± | 163  ± | 124.33± | 138  ± | 112  ± |
|  | 2.52^b^ | 1^b^ | 4.62^ab^ | 2.65^ab^ | 3.61^ab^ | 3^ab^ | 1.73^ab^ | 1^ab^ | 6.11^ab^ | 3^ab^ | 1^ab^ | 0.58^ab^ | 1.73^a^ | 1^a^ |
| 36.3 | 257  ± | 176  ± | 207.67± | 150.33± | 174  ± | 133.33± | 161.67± | 125.33± | 187.67± | 134.67± | 158  ± | 123.33± | 134  ± | 110.67± |
|  | 3^b^ | 1^b^ | 4.04^ab^ | 2.89^ab^ | 2.65^ab^ | 2.52^ab^ | 1.53^ab^ | 0.58^ab^ | 5.85^ab^ | 3.06^ab^ | 1^ab^ | 0.58^ab^ | 1.73^a^ | 1.52^a^ |
| 51.1 | 244  ± | 173  ± | 197  ± | 147.67± | 163.67± | 130.67± | 152.67± | 123  ± | 177.67± | 132.67± | 150  ±  1^ab^ | 121.67± | 127  ± | 109  ± |
|  | 3.61^b^ | 1^b^ | 3.61^ab^ | 2.31^ab^ | 1.53^ab^ | 2.08^ab^ | 1.5^ab^ | 1^ab^ | 4.93^ab^ | 3.06^ab^ |  | 1.54^ab^ | 1.72^a^ | 1^a^ |
| 71.9 | 22  3± | 168  ± | 179.67± | 143.33± | 148  ± | 126  ± | 140.33± | 120  ± | 162.67± | 130.33± | 138.33  ± | 118.33± | 117  ± | 106  ± |
|  | 5.29^b^ | 1^b^ | 3.51^ab^ | 2.08^ab^ | 1^ab^ | 1^ab^ | 3.06^ab^ | 2^ab^ | 4.93^ab^ | 3.51^ab^ | 0.58^ab^ | 0.58^ab^ | 1.74^a^ | 1^a^ |
| 101 | 192.67± | 160.33± | 155.67± | 137  ± | 128.33± | 120.67± | 122.33± | 115  ± | 142.3  ± | 126  ± | 121.33  ± | 113.33± | 103  ± | 102.33± |
|  | 7.37^b^ | 3.06^b^ | 4.16^ab^ | 2.65^ab^ | 2.31^ab^ | 1.53^ab^ | 3.06^ab^ | 2.65^ab^ | 3.79^ab^ | 3^ab^ | 0.57^ab^ | 1.54^ab^ | 1.7^a^ | 1.52^a^ |
| 142 | 155.67± | 150.67± | 127  ± | 129.67± | 104.67± | 113.33± | 100.67± | 108.67± | 116.67± | 119  ± | 100.1  ± | 107  ± | 85.07  ± | 96.63  ± |
|  | 7.02^b^ | 5.13^b^ | 3.61^ab^ | 3.21^ab^ | 2.82^ab^ | 1.53^ab^ | 3.21^ab^ | 2.52^ab^ | 3.21^ab^ | 3^ab^ | 0.78^ab^ | 1^ab^ | 1.25^a^ | 1^a^ |
| 200 | 116  ± | 139.33± | 96.23  ± | 119.67± | 79.17  ± | 104.33± | 76.4  ± | 99.67  ± | 87.83  ± | 109.33± | 75.97  ± | 98.77  ± | 65.03  ± | 88.73  ± |
|  | 5.57^b^ | 5.68^b^ | 2.66^ab^ | 3.21^ab^ | 1.59^ab^ | 1.53^ab^ | 2.74^ab^ | 2.52^ab^ | 2.11^ab^ | 2.08^ab^ | 0.46^ab^ | 0.31^ab^ | 0.97^a^ | 0.97^a^ |
