# Supplementary material for: Xylo‐oligosaccharides as texture modifier compounds in aqueous media and in combination with food thickeners
Source: Food Sci Nutr. 2019 Sep 10;8(7):3023–30. doi: 10.1002/fsn3.1177 (PMC7382132; doi:10.1002/fsn3.1177)
Supplement: Supplementary file 4 [file FSN3-8-3023-s004.docx]

Table S4. Storage and loss moduli values of xanthan gum gels prepared with or without xylo-oligosaccharides addition

|  | **0** | | **95P 1%** | | **70P 1%** | | **70L 1%** | | **95P 3%** | | **70P 3%** | | **70L 3%** | |
| --- | --- | --- | --- | --- | --- | --- | --- | --- | --- | --- | --- | --- | --- | --- |
| **Strain (%)** | G' | G" | G' | G" | G' | G" | G' | G" | G' | G" | G' | G" | G' | G" |
| 0.601 | 178.33  ±  17.62^b^ | 72.53  ±  8.55 | 170.67  ±  9.07^ab^ | 79.97  ±  2.63^b^ | 153.33  ±  7.23^ab^ | 62.6  ±  4.72^ab^ | 162  ±  13.23^ab^ | 69.67  ±  3.77 | 149.67  ±  8.74^ab^ | 63.433  ±  6.77^ab^ | 123.67  ±  12.01^ab^ | 51.13  ±  5.16^a^ | 140.33  ±  1.52^ab^ | 56.4  ±  0.85^ab^ |
| 0.847 | 170  ±  14.78^a^ | 62.33  ±  5.99^ab^ | 161.33  ±  8.5^a^ | 66.4  ±  1.45^b^ | 150  ±  7.81^a^ | 56  ±  3.21^ab^ | 156.33  ±  9.61^a^ | 60.27  ±  2.63^ab^ | 146.33  ±  6.66^ab^ | 57.3  ±  4.23^ab^ | 122.67  ±  10.5^a^ | 50.1  ±  3.39^a^ | 139  ±  1^a^ | 52.93  ±  0.81^ab^ |
| 1.19 | 162.33  ±  12.9^a^ | 53.3  ±  4.6^ab^ | 155.67  ±  8.33^a^ | 55.87  ±  1.19^b^ | 145  ±  9.54^a^ | 47.6  ±  3.08^ab^ | 151.33  ±  7.09^a^ | 51.03  ±  1.78^ab^ | 140  ±  5.67^a^ | 48.3  ±  3.29^ab^ | 118.67  ±  9.45^a^ | 42.93  ±  1.82^a^ | 134.67  ±  0.58^a^ | 45.2  ±  0.7^ab^ |
| 1.68 | 156.67  ±  12.5^a^ | 46.4  ±  3.39^ab^ | 152  ±  7.21^a^ | 48.37  ±  0.96^b^ | 141.33  ±  9.81^a^ | 41.43  ±  2.65^ab^ | 148.67  ±  6.35^a^ | 44.33  ±  1.33^ab^ | 136  ±  4^a^ | 41.67  ±  2.51^ab^ | 115.67  ±  10.12^a^ | 37.37  ±  1.83^a^ | 133.67  ±  2.89^a^ | 39.67  ±  0.1^ab^ |
| 2.36 | 153  ±  12.12^a^ | 41.73  ±  2.76^ab^ | 149.67  ±  6.66^a^ | 43.27  ±  09^b^ | 140.67  ±  8.38^a^ | 37.33  ±  2.14^ab^ | 146.67  ±  6.49^a^ | 39.63  ±  0.83^ab^ | 135  ±  3.61^a^ | 37.3  ±  2^ab^ | 115  ±  9.64^a^ | 33.4  ±  1.82^a^ | 133.33  ±  3.21^a^ | 35.6  ±  0.69^ab^ |
| 3.32 | 151  ±  10.82^a^ | 38.7  ±  2.56^ab^ | 148  ±  6.24^a^ | 39.7  ±  1.05^b^ | 140.67  ±  3.81^a^ | 34.57  ±  1.74^ab^ | 145.33  ±  7.77^a^ | 36.33  ±  0.61^ab^ | 135.67  ±  4.04^a^ | 34.73  ±  1.94^ab^ | 114.67  ±  8.74^a^ | 30.8  ±  2^a^ | 133  ±  1.73^a^ | 32.9  ±  0.61^ab^ |
| 4.67 | 148  ±  11.27^a^ | 36.17  ±  2^ab^ | 146  ±  5.29^a^ | 36.93  ±  1^b^ | 139.67  ±  5.86^a^ | 32.4  ±  1.76^ab^ | 144  ±  7.81^a^ | 34.2  ±  0.35^ab^ | 134.67  ±  3.06^a^ | 32.73  ±  1.8^ab^ | 114  ±  8.19^a^ | 28.8  ±  2.22^a^ | 133  ±  1.73^a^ | 31.17  ±  0.64^ab^ |
| 6.58 | 145.33  ±  10.97^a^ | 34.1  ±  1.49^ab^ | 144  ±  4.58^a^ | 34.73  ±  1.01^b^ | 139  ±  5.29^a^ | 30.9  ±  1.61^ab^ | 142.67  ±  8.14^a^ | 32.67  ±  0.35^ab^ | 134  ±  3^a^ | 31.43  ±  1.72^ab^ | 112.67  ±  7.51^a^ | 27.2  ±  1.99^a^ | 132.67  ±  1.15^a^ | 30  ±  0.61^ab^ |
| 9.26 | 142.67  ±  10.69^a^ | 32.27  ±  0.95^ab^ | 141.67  ±  4.16^a^ | 32.87  ±  0.81^b^ | 137.33  ±  4.04^a^ | 29.67  ±  1.37^ab^ | 140.67  ±  8.96^a^ | 31.37  ±  0.61^ab^ | 132.67  ±  2.52^a^ | 30.5  ±  1.51^ab^ | 111.67  ±  6.03^a^ | 25.9  ±  1.76^a^ | 131.67  ±  2.08^a^ | 29.17  ±  0.47^ab^ |
| 13 | 140  ±  10.39^a^ | 31.03  ±  0.93^ab^ | 139  ±  3.61^a^ | 31.5  ±  0.61^b^ | 135.33  ±  3.51^a^ | 28.77  ±  0.95^ab^ | 137.67  ±  8.96^a^ | 30.3  ±  1.08^ab^ | 131  ±  2^a^ | 29.73  ±  1.25^ab^ | 110.33  ±  5.51^a^ | 24.9  ±  1.41^a^ | 130  ±  1.73^a^ | 28.43  ±  0.32^ab^ |
| 18.3 | 135.67  ±  9.81^a^ | 30.43  ±  1.01^ab^ | 134.67  ±  3.21^a^ | 30.83  ±  0.68^b^ | 131.33  ±  3.21^a^ | 28.43  ±  0.67^ab^ | 133.33  ±  8.39^a^ | 29.87  ±  1.51^ab^ | 127.67  ±  1.53^a^ | 29  ±  0.95^ab^ | 108.67  ±  5.03^a^ | 24.13  ±  1.17^a^ | 126.67  ±  2.08^a^ | 27.97  ±  0.21^ab^ |
| 25.8 | 127.67  ±  9.87^a^ | 31.4  ±  1.4^ab^ | 126.33  ±  3.06^a^ | 31.7  ±  1^b^ | 124  ±  2.65^a^ | 29.6  ±  0.66^ab^ | 125  ±  7.81^a^ | 30.93  ±  2.06^ab^ | 121.67  ±  0.58^a^ | 29.07  ±  0.85^ab^ | 104.27  ±  4.61^a^ | 24.13  ±  1.03^a^ | 121  ±  2^a^ | 28.1  ±  0.3^ab^ |
| 36.3 | 111.33  ±  7.57^a^ | 36.47  ±  2.42^a^ | 111.33  ±  2.08^a^ | 36.1  ±  1.61^a^ | 109  ±  1.73^a^ | 34.57  ±  1.29^a^ | 109.67  ±  5.51^a^ | 35.6  ±  2.87^a^ | 110.33  ±  0.58^a^ | 31.47  ±  0.95^a^ | 95.57  ±  3.95^a^ | 25.83  ±  1^a^ | 109.67  ±  1.53^a^ | 30.73  ±  0.51^a^ |
| 51.1 | 85.63  ±  4.54^ab^ | 44.23  ±  3.63^a^ | 86.23  ±  0.74^ab^ | 43.37  ±  1.85^a^ | 83.7  ±  1^ab^ | 42.2  ±  1.59^a^ | 84.26  ±  3.67^ab^ | 42.9  ±  3.49^a^ | 89.87  ±  0.23^b^ | 37.67  ±  0.76^a^ | 79.1  ±  2.71^a^ | 31.07  ±  1.39^a^ | 88.53  ±  0.83^ab^ | 37.87  ±  0.64^a^ |
| 71.9 | 57.5  ±  2.2^a^ | 47  ±  3.55^a^ | 58.67  ±  0.64^a^ | 46.3  ±  1.35^a^ | 56.6  ±  0.61^a^ | 45.1  ±  1.23^a^ | 56.9  ±  2.17^a^ | 45.53  ±  3.29^a^ | 63.47  ±  0.29^a^ | 42.77  ±  0.6^a^ | 56.57  ±  1.04^a^ | 36.27  ±  1.88^a^ | 61.17  ±  1.7^a^ | 43.3  ±  0.36^a^ |
| 101 | 35.8  ±  1.01^a^ | 42.97  ±  2.89^a^ | 36.8  ±  0.61^a^ | 42.63  ±  0.86^a^ | 35.33  ±  0.55^a^ | 41.47  ±  0.74^a^ | 35.5  ±  1.22^a^ | 41.73  ±  2.86^a^ | 40.27  ±  0.47^a^ | 41.47  ±  0.45^a^ | 36.23  ±  0.25^a^ | 35.73  ±  1.6^a^ | 38.1  ±  0.4^a^ | 41.43  ±  0.15^a^ |
| 142 | 21.77  ±  0.42^a^ | 35.73  ±  2.14^a^ | 22.37  ±  0.55^a^ | 35.73  ±  0.57^a^ | 21.47  ±  0.41^a^ | 34.67  ±  0.47^a^ | 21.47  ±  0.67^a^ | 34.7  ±  2.04^a^ | 24.4  ±  0.36^a^ | 35.67  ±  0.32^a^ | 22.1  ±  0.1^a^ | 30.93  ±  1.05^a^ | 22.8  ±  01^a^ | 35.2  ±  0.1^a^ |
| 200 | 13.33  ±  0.31^a^ | 28.37  ±  1.59^a^ | 13.67  ±  0.38^a^ | 28.47  ±  0.4^a^ | 13.2  ±  0.26^a^ | 27.6  ±  0.36^a^ | 13.17  ±  0.5^a^ | 27.7  ±  1.48^a^ | 14.63  ±  0.23^a^ | 28.67  ±  0.32^a^ | 13.33  ±  0.06^a^ | 25  ±  0.7^a^ | 13.6  ±  01^a^ | 281  ±  0.1^a^ |
