# Supplementary material for: Xylo‐oligosaccharides as texture modifier compounds in aqueous media and in combination with food thickeners
Source: Food Sci Nutr. 2019 Sep 10;8(7):3023–30. doi: 10.1002/fsn3.1177 (PMC7382132; doi:10.1002/fsn3.1177)
Supplement: Supplementary file 5 [file FSN3-8-3023-s005.docx]

Table S5. Storage and loss moduli values of locust bean gum + xanthan gum gels prepared with or without xylo-oligosaccharides addition

|  | 0% | | 95P 1% | | 70P 1% | | 70L 1% | | 95P 3% | | 70P 3% | | 70L 3% | |
| --- | --- | --- | --- | --- | --- | --- | --- | --- | --- | --- | --- | --- | --- | --- |
| Strain (%) | G' | G" | G' | G" | G' | G" | G' | G" | G' | G" | G' | G" | G' | G" |
| 0.0791 | 1756.67 | 279 | 1663.33 | 428.67 | 1530 | 414.67 | 1286.67 | 316 | 1570 | 302.67 | 1500 | 387 | 1282.5 | 290.25 |
|  | ± | ± | ± | ± | ± | ± | ± | ± | ± | ± | ± | ± | ± | ± |
|  | 120.14^b^ | 6.56^b^ | 185.83^ab^ | 63.9^ab^ | 70^ab^ | 27.32^ab^ | 90.74^a^ | 43.09^a^ | 88.88^ab^ | 22.9^ab^ | 55.68^ab^ | 70.17^ab^ | 72.74^a^ | 99.13^ab^ |
| 0.109 | 1616.67 | 260.33 | 1490 | 370.33 | 1363.33 | 354 | 1226.67 | 296.67 | 1466.67 | 275 | 1340 | 337.67 | 1210 | 281.75 |
|  | ± | ± | ± | ± | ± | ± | ± | ± | ± | ± | ± | ± | ± | ± |
|  | 145.03^a^ | 8.144^a^ | 160.94^a^ | 50.24^a^ | 80.83^a^ | 23.43^a^ | 76.38^a^ | 36.47^a^ | 75.06^a^ | 15.62^a^ | 45.83^a^ | 51.69^a^ | 33.67^a^ | 52.65^a^ |
| 0.154 | 1543.33 | 227.67 | 1376.67 | 310 | 1263.33 | 301.67 | 1116.67 | 266 | 1386.67 | 241.67 | 1246.67 | 292 | 1112.5 | 248.25 |
|  | ± | ± | ± | ± | ± | ± | ± | ± | ± | ± | ± | ± | ± | ± |
|  | 160.1^b^ | 3.21^a^ | 145.72^ab^ | 35.51^a^ | 77.67^ab^ | 31.53^a^ | 66.58^ab^ | 29.51^a^ | 80.83^ab^ | 4.62^a^ | 50.33^ab^ | 45.57^a^ | 53.15^a^ | 37.23^a^ |
| 0.216 | 1500 | 201 | 1313.33 | 258 | 1203.33 | 253.67 | 1056.67 | 221.67 | 1330 | 210.33 | 1190 | 246 | 1060.5 | 213.75 |
|  | ± | ± | ± | ± | ± | ± | ± | ± | ± | ± | ± | ± | ± | ± |
|  | 170^a^ | 10.44^a^ | 137.96^a^ | 27.22^a^ | 70.95^a^ | 23.07^a^ | 50.33^a^ | 18.5^a^ | 81.85^a^ | 2.31^a^ | 50^a^ | 33.05^a^ | 66.54^a^ | 25.66^a^ |
| 0.304 | 1473.33 | 171.67 | 1270 | 226.33 | 1163.33 | 213 | 1014.67 | 193.33 | 1290 | 181 | 1150 | 208 | 1024.75 | 184.25 |
|  | ± | ± | ± | ± | ± | ± | ± | ± | ± | ± | ± | ± | ± | ± |
|  | 1640.1^b^ | 6.66^a^ | 140^ab^ | 18.93^b^ | 68.07^ab^ | 13.08^ab^ | 43.18^b^ | 15.7^ab^ | 85.44^ab^ | 2.65^ab^ | 45.83^ab^ | 19.52^ab^ | 68^a^ | 18.71^ab^ |
| 0.428 | 1456.67 | 152.67 | 1240 | 195.67 | 1133.33 | 184 | 993.67 | 170 | 1273.33 | 159.67 | 1116.67 | 180.33 | 1004 | 163 |
|  | ± | ± | ± | ± | ± | ± | ± | ± | ± | ± | ± | ± | ± | ± |
|  | 155.03^b^ | 9.29^a^ | 130.78^ab^ | 15.89^a^ | 68.07^ab^ | 8.89^a^ | 33.86^a^ | 16.09^a^ | 80.81^ab^ | 2.31^a^ | 40.41^ab^ | 12.34^a^ | 72.79^ab^ | 18.94^a^ |
| 0.603 | 1456.67 | 140 | 1230 | 176.67 | 1113.33 | 164.6 | 987 | 154.667 | 1260 | 146.33 | 1093.33 | 161 | 982 | 146.25 |
|  | ± | ± | ± | ± | ± | ± | ± | ± | ± | ± | ± | ± | ± | ± |
|  | 145.03^b^ | 10.58^a^ | 121.66^ab^ | 11.55^a^ | 68.07^ab^ | 6.03^a^ | 21.66^a^ | 16.61^a^ | 70^ab^ | 2.89^a^ | 35.12^ab^ | 9.17^a^ | 64.6^a^ | 13.25^a^ |
| 0.849 | 1460 | 136.67 | 1220 | 165.33 | 1103.33 | 151 | 991 | 144.33 | 1250 | 135.67 | 1080 | 147.33 | 962.25 | 133.75 |
|  | ± | ± | ± | ± | ± | ± | ± | ± | ± | ± | ± | ± | ± | ± |
|  | 125.3^b^ | 13.32^a^ | 104.1^ab^ | 7.51^a^ | 61.1^ab^ | 5.57^a^ | 9.64^a^ | 10.69^a^ | 60^ab^ | 3.51^a^ | 26.46^ab^ | 7.77^a^ | 57.91^a^ | 11.47^a^ |
| 19.jan | 1466.67 | 135 | 1220 | 156 | 1096.66 | 140.33 | 998.67 | 137 | 1246.67 | 129.67 | 1070 | 137.33 | 945 | 124.75 |
|  | ± | ± | ± | ± | ± | ± | ± | ± | ± | ± | ± | ± | ± | ± |
|  | 105.9^b^ | 7.93^a^ | 79.37^ab^ | 4.36^a^ | 61.1^ab^ | 7.51^a^ | 21.01^a^ | 7.55^a^ | 50.33^ab^ | 3.21^a^ | 40^ab^ | 9.29^a^ | 45.73^a^ | 10.18^a^ |
| 1.68 | 1450 | 135.67 | 1216.67 | 152 | 1093.33 | 136.67 | 999.6 | 132.33 | 1233.33 | 126.67 | 1063.33 | 132.67 | 939.75 | 119.5 |
|  | ± | ± | ± | ± | ± | ± | ± | ± | ± | ± | ± | ± | ± | ± |
|  | 101.49^b^ | 11.68^a^ | 70.24^ab^ | 5.57^a^ | 64.29^ab^ | 8.08^a^ | 30.5^ab^ | 6.35^a^ | 40.41^ab^ | 4.93^a^ | 51.31^ab^ | 9.87^a^ | 45.91^a^ | 9.88^a^ |
| 2.36 | 1426.67 | 152 | 1210 | 149.33 | 1080 | 134.33 | 990.33 | 129.33 | 1216.67 | 127 | 1053.33 | 130.33 | 933.25 | 116.75 |
|  | ± | ± | ± | ± | ± | ± | ± | ± | ± | ± | ± | ± | ± | ± |
|  | 96.09^b^ | 21^a^ | 65.57^ab^ | 5.89^a^ | 55.68^ab^ | 7.64^a^ | 23.03^ab^ | 6.35^a^ | 45.09^ab^ | 7.81^a^ | 35.12^ab^ | 9.29^a^ | 42.32^a^ | 8.73^a^ |
| 3.32 | 1393.33 | 178.67 | 1190 | 152.67 | 1063.33 | 136.67 | 978.33 | 127.6 | 1190 | 131.67 | 1040 | 132 | 921 | 116 |
|  | ± | ± | ± | ± | ± | ± | ± | ± | ± | ± | ± | ± | ± | ± |
|  | 90.74^b^ | 27.65^b^ | 72.11^ab^ | 8.14^ab^ | 55.08^ab^ | 8.39^ab^ | 19.66^ab^ | 6.66^ab^ | 45.83^ab^ | 13.32^ab^ | 26.46^ab^ | 8.66^ab^ | 40.76^a^ | 8.08^a^ |
| 4.68 | 1363.33 | 221 | 1180 | 173.33 | 1037.33 | 149.67 | 985 | 143.67 | 1163.33 | 145.67 | 1014 | 141.33 | 916.25 | 124.5 |
|  | ± | ± | ± | ± | ± | ± | ± | ± | ± | ± | ± | ± | ± | ± |
|  | 90.74^b^ | 36.39^b^ | 70^ab^ | 15.31^ab^ | 54.49^ab^ | 11.85^ab^ | 51.86^ab^ | 27.32^ab^ | 32.15^ab^ | 21.22^ab^ | 29.46^ab^ | 19.8^ab^ | 58.64^a^ | 18.23^a^ |
| 6.59 | 1320 | 272.67 | 1126.67 | 199.33 | 994.66 | 175.67 | 949.67 | 155.33 | 1120 | 170.67 | 974.67 | 163 | 890.75 | 137.5 |
|  | ± | ± | ± | ± | ± | ± | ± | ± | ± | ± | ± | ± | ± | ± |
|  | 90^b^ | 41.65^a^ | 6.06^ab^ | 10.12^a^ | 53.15^ab^ | 16.2^a^ | 44.66^ab^ | 34.59^a^ | 20^ab^ | 32.15^a^ | 26.56^ab^ | 20.66^a^ | 56.83^a^ | 20.4^a^ |
| 28.szept | 1280 | 325.33 | 1073.33 | 240 | 934.33 | 214.33 | 898.33 | 180.67 | 1086.67 | 213.67 | 914 | 198.67 | 845.25 | 162.75 |
|  | ± | ± | ± | ± | ± | ± | ± | ± | ± | ± | ± | ± | ± | ± |
|  | 85.44^b^ | 42.71^a^ | 55.08^ab^ | 14.93^a^ | 59.5^ab^ | 14.36^a^ | 40.2^b^ | 48.06^a^ | 15.28^ab^ | 46.69^a^ | 27.84^ab^ | 26^a^ | 50.41^a^ | 25^a^ |
| 13.jan | 1223.33 | 374 | 1010.33 | 283.67 | 861 | 254 | 830.33 | 211.33 | 1030 | 251.67 | 838.67 | 237.67 | 786.75 | 197.25 |
|  | ± | ± | ± | ± | ± | ± | ± | ± | ± | ± | ± | ± | ± | ± |
|  | 86.22^b^ | 45^b^ | 39.5^ab^ | 11.55^ab^ | 65.2^ab^ | 9.85^ab^ | 38.37^ab^ | 57.14^ab^ | 10^ab^ | 44.27^ab^ | 28.1^ab^ | 27.39^ab^ | 43.76^a^ | 25.82^a^ |
| 18.ápr | 1153.33 | 420 | 918.33 | 312.67 | 776.33 | 280.67 | 750.33 | 239.33 | 964.67 | 276.33 | 751.33 | 266.33 | 716.5 | 227.25 |
|  | ± | ± | ± | ± | ± | ± | ± | ± | ± | ± | ± | ± | ± | ± |
|  | 83.27^a^ | 56.63^b^ | 33.13^a^ | 19.5^ab^ | 68.39^a^ | 6.81^ab^ | 32.08^a^ | 52.6^ab^ | 7.23^a^ | 33.5^ab^ | 25.54^a^ | 23.71^ab^ | 36.86^a^ | 24.45^a^ |
| 26 | 1062.67 | 458.67 | 804.33 | 343.67 | 690 | 312 | 657.67 | 262 | 887.67 | 294.33 | 658.67 | 294.33 | 634.5 | 247.25 |
|  | ± | ± | ± | ± | ± | ± | ± | ± | ± | ± | ± | ± | ± | ± |
|  | 82.59^a^ | 68.04^b^ | 28.29^a^ | 26.5^ab^ | 73.82^a^ | 4.58^ab^ | 24.01^a^ | 37.16^ab^ | 8.96^a^ | 25.01^ab^ | 20.11^a^ | 27.3^ab^ | 30.23^a^ | 20.93^a^ |
| febr.36 | 966.33 | 482.67 | 694.33 | 366 | 583.33 | 325.33 | 563.67 | 287 | 788.67 | 330.67 | 557 | 304 | 542.75 | 260.75 |
|  | ± | ± | ± | ± | ± | ± | ± | ± | ± | ± | ± | ± | ± | ± |
|  | 76.29^a^ | 65.31^b^ | 24.5^a^ | 22.61^ab^ | 67.31^a^ | 13.58^ab^ | 18.01^a^ | 19^ab^ | 19.14^a^ | 16.5^ab^ | 21.79^a^ | 23.39^ab^ | 24.51^a^ | 20.29^a^ |
| márc.51 | 871 | 496.3 | 585 | 363.67 | 486.66 | 322.67 | 475 | 294 | 664.33 | 356.33 | 467.67 | 308.67 | 455.75 | 272 |
|  | ± | ± | ± | ± | ± | ± | ± | ± | ± | ± | ± | ± | ± | ± |
|  | 71.39^a^ | 60.58^b^ | 23.3^a^ | 17.01^ab^ | 54^a^ | 8.96^ab^ | 19.05^a^ | 22.72^ab^ | 24.44^a^ | 12.06^ab^ | 21.13^a^ | 15.31^ab^ | 22.32^a^ | 12.41^a^ |
| febr.72 | 769.67 | 499.67 | 487.67 | 345.33 | 403 | 303 | 383.33 | 281.67 | 552 | 355.33 | 388.67 | 292.67 | 373.75 | 263.25 |
|  | ± | ± | ± | ± | ± | ± | ± | ± | ± | ± | ± | ± | ± | ± |
|  | 68.64^a^ | 53^b^ | 23.46^a^ | 20.65^ab^ | 45.04^a^ | 11.38^ab^ | 14.19^a^ | 22.68^ab^ | 30.34^a^ | 11.37^ab^ | 20.23^a^ | 6.81^ab^ | 18.19^a^ | 11.95^a^ |
| 101 | 658.67 | 491.67 | 395 | 320 | 324.67 | 276.67 | 302 | 256.33 | 441.67 | 342.33 | 315.33 | 267.67 | 299.75 | 243.25 |
|  | ± | ± | ± | ± | ± | ± | ± | ± | ± | ± | ± | ± | ± | ± |
|  | 84.72^a^ | 38^b^ | 25.36^a^ | 18.52^ab^ | 37.53^a^ | 10.69^ab^ | 9.54^a^ | 19.86^ab^ | 39.37^a^ | 11.37^ab^ | 21.36^a^ | 4.93^ab^ | 15.41^a^ | 11.53^a^ |
| 143 | 536.67 | 478.33 | 300.67 | 285 | 248.67 | 254.33 | 222 | 224.67 | 328.67 | 320 | 245.33 | 246.67 | 230.25 | 218 |
|  | ± | ± | ± | ± | ± | ± | ± | ± | ± | ± | ± | ± | ± | ± |
|  | 125.38^b^ | 24.11^b^ | 21.39^ab^ | 16^ab^ | 29.77^ab^ | 6.03^ab^ | 8.19^a^ | 16.29^a^ | 42.91^ab^ | 11.14^ab^ | 24.01^ab^ | 9.07^ab^ | 12.47^ab^ | 10.86^a^ |
| 201 | 422.33 | 460 | 210.33 | 248.33 | 178.33 | 215.33 | 150.33 | 192.33 | 219.67 | 287.33 | 178.33 | 214 | 163 | 187.25 |
|  | ± | ± | ± | ± | ± | ± | ± | ± | ± | ± | ± | ± | ± | ± |
|  | 141.85^b^ | 45.83^b^ | 18.5^ab^ | 15.57^ab^ | 22.28^ab^ | 6.11^ab^ | 7.57^a^ | 16.07^a^ | 35.92^ab^ | 5.51^ab^ | 22.28^ab^ | 4^ab^ | 11.34^ab^ | 9.18^a^ |
